# Dual-stimuli responsive ionophore for OFF–ON–OFF transmembrane calcium ion transport and inter-vesicle signalling†

**DOI:** 10.1039/d6sc02506d

**Published:** 2026-07-01

**Authors:** Gurshinder Kaur, Krzysztof M. Bąk, Daniel C. Edwards, Scott L. Cockroft, Mathew H. Horrocks, Matthew J. Langton

**Affiliations:** a Chemistry Research Laboratory, Department of Chemistry, University of Oxford 12 Mansfield Road Oxford OX1 3TA UK matthew.langton@chem.ox.ac.uk; b EaStCHEM School of Chemistry, University of Edinburgh Joseph Black Building, David Brewster Rd Edinburgh EH9 3FJ UK; c IRR Chemistry Hub, Institute of Regeneration and Repair, University of Edinburgh Edinburgh EH16 4UU UK

## Abstract

The design of stimuli-responsive synthetic ion transporters is inspired by natural protein channels and pumps, which regulates transport by responding to changes in extracellular stimuli such as light, membrane potential and small molecules. Examples of synthetic calcium transporters are rare, despite the widespread importance of calcium in various biological processes including signalling, nerve transmission and as enzyme cofactors. Herein we report a dual-stimuli responsive ionophore able to mediate transmembrane calcium ion transport across lipid bilayer membranes, with transport sequentially activated and deactivated by light and enzymatic stimuli. We exploit this system to achieve inter-vesicle signal-induced calcium transport, in which calcium influx to one population of vesicles is distally activated *via* signalling from neighbouring vesicles in a manner reminiscent of signalling pathways in nature.

## Abstract 2

A dual-stimulus responsive ion carrier capable of photo-triggered transmembrane calcium ion transport activation, and enzymatic deactivation. The system enables induced calcium transport *via* signalling between populations of synthetic cells.

## Graphical abstract

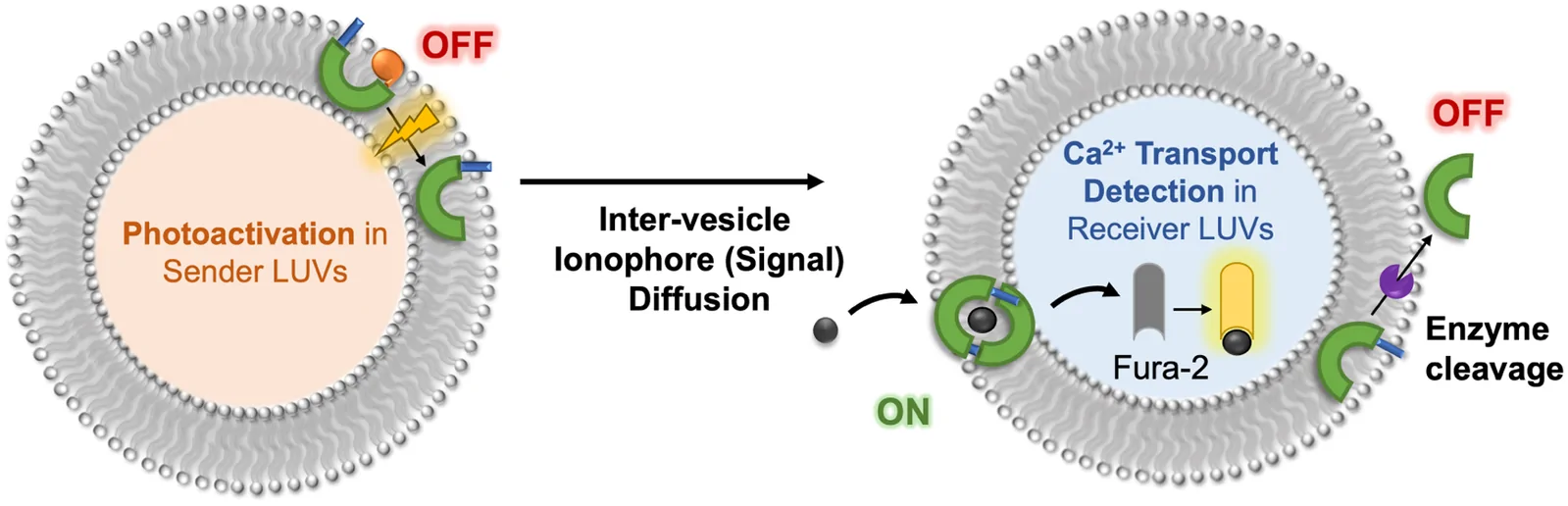

## Introduction

In nature, a multitude of essential protein channels and pumps precisely and selectively control the movement of ions and molecules across the lipid bilayer membrane of cells. These respond to local physical and chemical changes by regulating their activity accordingly.^1^ Inspired by these naturally occurring transporters, there has been a recent drive to develop stimuli-responsive small molecules that mimic nature's transport mechanisms for a range of applications, such as targeted therapeutics for channelopathies and cancer, new ion sensing techniques and tools for use in synthetic biology.^2–9^ Incorporating responsive moieties into the transporter's design to regulate activity, such as groups responsive to light,^10–18^ redox,^19–21^ enzymes,^22,23^ enables spatio-temporal control over the transport process.

Calcium ions (Ca^2+^) are involved in a wide range of biological functions, such as being the signalling agent in muscle contraction, in neural communication, in cell apoptosis and in the regulation of enzymes.^24–27^ Mis-regulation of calcium channels has been implicated in a range of neurological disorders, including chronic pain, epilepsy and migraine.^24,28^ However, despite this importance of transmembrane calcium transport, only a handful of examples of artificial Ca^2+^ transporters that operate in lipid bilayers have been reported. These include a pyridyl–triazole peptidic mobile carrier reported by Saha *et al.*, and an *o*-phenanthroline-oxadiazole-based pentamer scaffold by Lin *et al.*^29,30^ Elie *et al.* reported a benzimidazolium-based channel that promotes Ca^2+^/2Cl^−^ symport in *E. coli* strains, showcasing the potential of ionophores functioning in bacterial membranes as antibiotics,^31^ while Kawano *et al.* have reported metal–organic cuboctahedra-based Ca^2+^ channels.^32^ In contrast to naturally occurring Ca^2+^ mobile carriers like ionomycin, calcimycin or lasalocid A that operate by electroneutral mechanisms,^33–35^ Wang *et al.* have reported an ionophore ETH-129 that works *via* an electrogenic mechanism and exhibited Ca^2+^ transport activity in yeast mitochondria.^36^ However, this handful of reported calcium ionophores are not stimuli-responsive and, once introduced to a bilayer, they transport ions until the calcium gradient is dissipated. Photo-regulated Ca^2+^ ionophores would allow for a broader range of applications by enabling calcium transport to be externally triggered with spatio-temporal control and hence enable regulation of the downstream biology. This promise has motivated recent efforts to engineer Ca^2+^-permeable channelrhodopsins,^37^ with emerging applications in the optogenetics optical control over Ca^2+^ flux in neural tissues.^38–40^ Photo-responsive small molecule Ca^2+^ transporters would therefore be an attractive, readily accessible and scalable chemical tool to complement such protein-based systems but are, to the best of our knowledge, unprecedented.

**Fig. 1:**
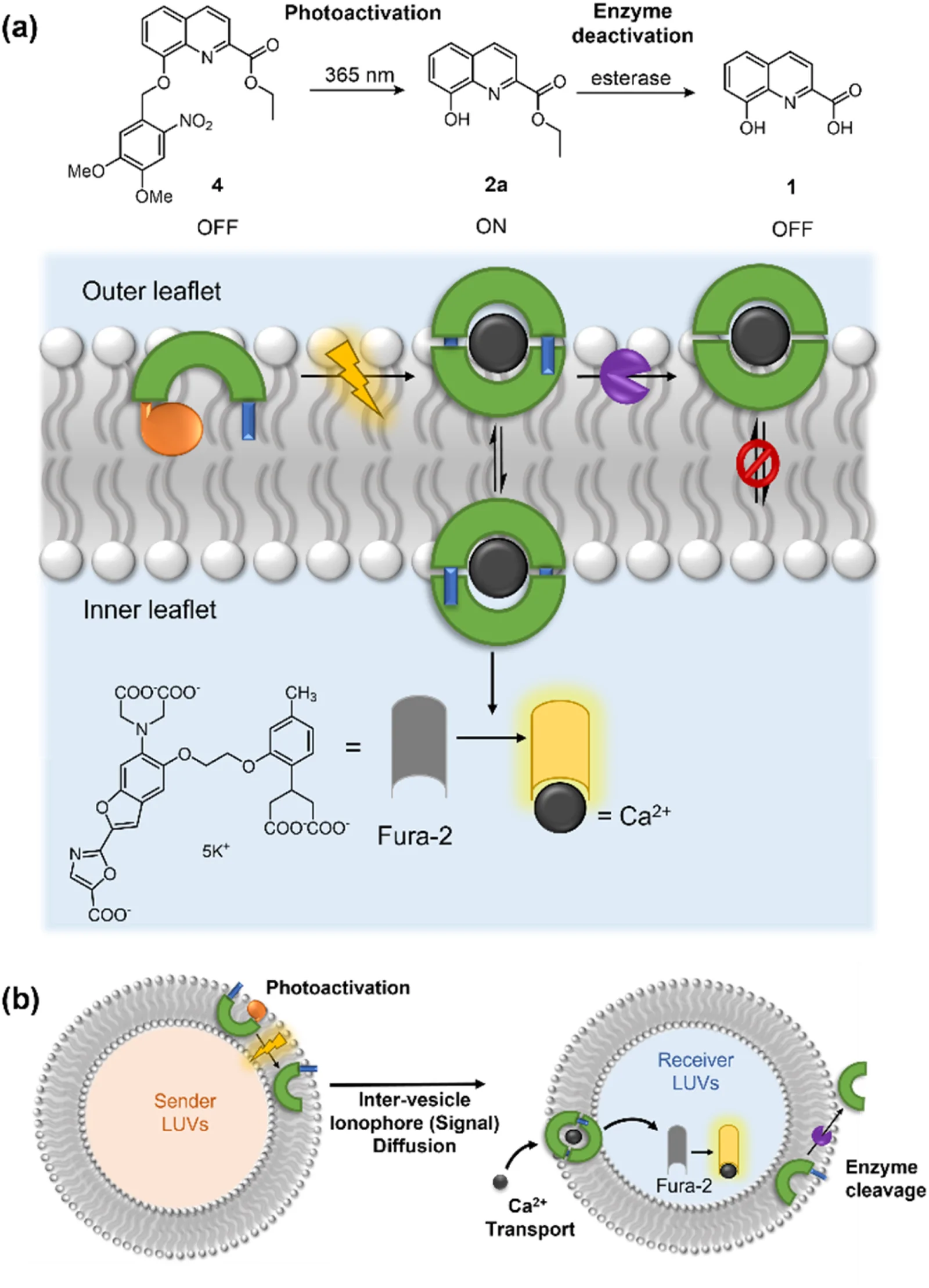
(a) Chemical structures of photo-caged cationophore 4, calcium ionophore 2a, inactive pro-ionophore 1 and calcium-sensitive fluorophore Fura-2, and schematic representation of photoactivation and enzymatic deactivation of transmembrane Ca^2+^ transport; (b) schematic representation of inter-vesicle signalling-induced calcium transport.

Herein, we report the first example of a photo-responsive synthetic calcium ionophore. We demonstrate that by photo-caging a 2-carboxy-8-hydroxyquinoline ester derivative, Ca^2+^ transport can be inhibited, and then reinstated under photoirradiation. An additional level of control can be achieved by using an esterase enzyme to enable an overall OFF–ON–OFF switching of calcium transport (Fig. 1a). We also demonstrate that this system can be used to signal between different populations of vesicles, triggering Ca^2+^ influx in a manner reminiscent of biological Ca^2+^ signalling (Fig. 1b).

## Results and discussion

### Design of a Ca^2+^ transporter and transport experiments in LUVs

Previously we have reported photo-caged variants of the hydroxyquinoline derivative clioquinol able to photo-regulate zinc transport and signal between vesicles.^14^ We envisaged employing a similar approach here to target calcium transport, and we identified 8-hydroxyquinoline-2-carboxylic acid 1 as the core scaffold for the target Ca^2+^ ionophore. This was also inspired by naturally occurring derivatives such as calcimycin (A23187) which act as an anionic, tridentate Ca^2+^ ionophores.^35,41^ Esterification of 1 enables tuning of lipophilicity and facilitates uptake into the lipid bilayer, while deprotonation of the hydroxyquinoline phenol upon complexation generates a tridentate monoanionic ester that was expected to form a neutral 2 : 1 ionophore-Ca^2+^ complex capable of diffusing across lipid bilayers. Electroneutrality of the transport process was expected to be achieved by antiport of either protons or sodium cations from the buffer. Accordingly, we prepared the ethyl (2a) and hexyl (2b) ester derivatives of 1. Full synthetic procedures and characterisation for all synthesised compounds are available in the SI.

The Ca^2+^ transport activities of ionophores 1, 2a, 2b were studied using fluorescence assays in large unilamellar vesicles (LUVs) containing the calcium-sensitive fluorophore Fura-2. Fura-2 acts as a ratiometric turn-on fluorescent probe for a range of divalent cations (*λ*_ex_ = 340/380 nm, *λ*_em_ = 510 nm), including Ca^2+^ ions.^42,43^ LUVs with an average diameter of 200 nm were prepared from palmitoyl-2-oleoyl-*sn*-glycero-3-phosphocholine (POPC) containing Fura-2 (314 µM), in 100 mM NaCl buffered to pH 7 using 10 mM HEPES. A pulse of 100 µM CaCl_2_ was added to the LUVs solution to generate a transmembrane calcium ion gradient. Dissipation of this gradient by the ionophore was reported *via* changes in the ratiometric Fura-2 emission intensity, which is correlated to the increasing concentration of Ca^2+^ ions in the lumen of the vesicles. To calibrate the assay to 100% transport, a pulse of calcimycin was added at the end of the experiment to fully dissipate the gradient.

**Fig. 2:**
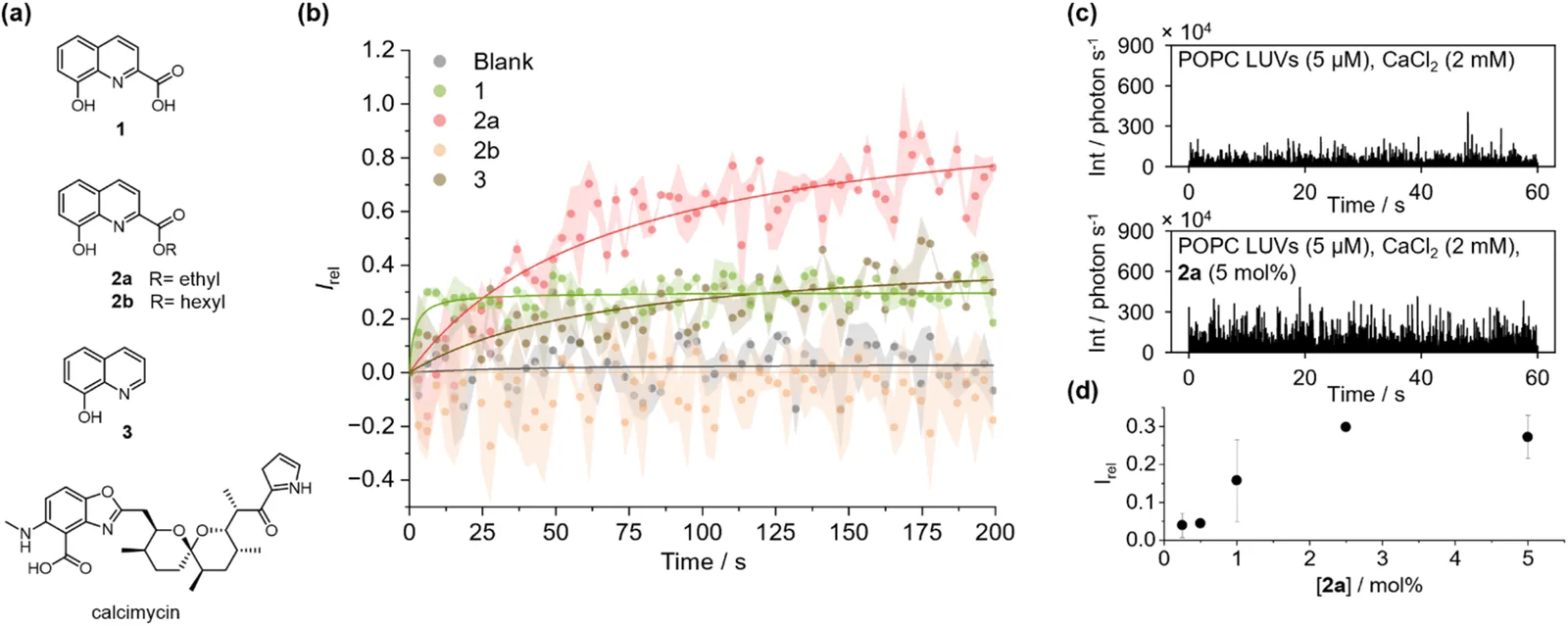
(a) Quinoline-based ligands investigated for transmembrane Ca^2+^ ion transport; (b) change in ratiometric Fura-2 emission, *I*_rel_ (*λ*_ex_ = 340/380 nm, *λ*_em_ = 510 nm) induced by Ca^2+^ transport upon the addition of 1, 2a, 2b and 3 (2.5 µM) in DMSO to POPC LUVs (80 µM) containing 314 µM Fura-2, 100 mM internal and external NaCl, 10 µM internal EDTA and 100 µM external Ca^2+^, all buffered with 10 mM HEPES to pH 7.0; lines represent fit to a first order rate equation; shaded regions represent standard deviations; (c) representative single-liposome calcium transport studies with 2a. Example of data collected over one minute for Fluo-8 dye-filled LUVs in the presence of CaCl_2_ alone (top) and in the presence of 5 mol% (relative to POPC) 2a (bottom); (d) activity of compound 2a, at various concentrations, in a single liposome calcium transport assay.

The Fura-2 fluorescence Ca^2+^ transport assay results for 1, 2a, 2b and 3 are shown in Fig. 2b. Maximum activity was observed for ethyl ester 2a, whilst for 1, minimal activity was observed. This suggests that, as expected, the anionic carboxylate is detrimental to transport, likely due to its hydrophilicity and hence inability to cross the membrane. Hexyl ester 2b (*c* log *P* = 4.4 *vs.* 2.6 for compound 2a) was completely inactive, presumably due to its increased lipophilicity inhibiting delivery and mobility within the membrane, as previously observed for anion transporters.^44^ The bidentate ligand 8-hydroxyquinoline 3 (*c* log *P* = 1.9), which lacks the ester group, exhibited poor transport, highlighting that a tridentate ionophore is essential for the transport of Ca^2+^ ions. Analysis of the concentration dependence of Ca^2+^ transport activity of ionophore 2a revealed increasing activity with ionophore concentration (Fig. S8b). Fitting the corresponding dose–response curve to the Hill equation afforded an EC_50_ value of 1.5 ± 0.6 µM (Fig. S8c). Analysis of the initial rates of transport revealed a dependence on the square of ionophore concentration, which is indicative of a 2 : 1 ligand : cation complex involved in the transport (Fig. S8d). This is consistent with the expected formation of a neutral, membrane-permeable 2 : 1 ionophore-Ca^2+^ complex, analogous to that observed for calcimycin. UV-vis titration experiments with 2a further validated this proposed transport stoichiometry, suggesting the formation of a 2 : 1 complex in methanol, with stepwise association constants too high to be determined (>10^7^ M^−1^). Comparable binding behaviour of 1, despite its negligible transport activity, supports the hypothesis that transport if inhibited by the hydrophilicity of the carboxylate group , rather than low affinity for Ca^2+^ (Fig. S5 and S6).

### Single-molecule liposome and ISE assay

The ability of 2a to mediate Ca^2+^ transport was further confirmed using a single-molecule liposome assay.^45^ Briefly, single-molecule confocal microscopy combined with fast-flow microfluidics enabled detection of fluorescence bursts from individual dye-loaded vesicles as they transited through a femtolitre confocal volume. Dissipation of Ca^2+^ gradient by 2a was monitored using LUVs containing Fluo-8 (10 µM), a selective turn-on calcium-binding dye (*λ*_ex_ = 488 nm). Addition of 2a (5 mol% relative to POPC) and calcium chloride (2 mM) to HEPES buffered LUVs suspension led to a notable increase in the intensity of fluorescence bursts (emission range: 525–540 nm) following a 5 minute incubation (Fig. 2c, d and S23). Importantly, single-vesicle measurements confirmed that the fluorescence increase arose from calcium transport into the vesicle, rather than vesicle rupture and dye leakage that would be obscured in bulk assays. These findings using fluorescent methods were independently corroborated using a Ca^2+^-selective electrode, which revealed calcium efflux from LUVs loaded with buffered CaCl_2_ solution, suspended in NaCl solution, in the presence of 2a (Fig. S21).

### Mechanistic transport studies

To avoid the build-up of a membrane potential, the transport of ions must occur either with the antiport of a like-charged ion, or co-transport (symport) of an oppositely charged ion. To investigate the mechanism of calcium transport by 2a, the Fura-2 assay was conducted in the presence of carbonyl cyaninde-*p*-trifluoromethoxyphenylhydrazone (FCCP), a weak acid protonophore. This revealed no significant change on the activity of 2a (Fig. S9). In the presence of gramicidin, an effective Na^+^ transporter, the transport rate was also unaffected (Fig. S12). Experiments in the presence of KCl revealed similar activities to those observed in the presence of NaCl, whilst the addition of valinomycin – an effective K^+^ transporter – did not significantly alter the observed activity (Fig. S10). These experiments suggest that Ca^2+^ transport activity is independent of the rate of any potential H^+^, Na^+^ or K^+^ transport.

The ability of 2a to transport protons *via* Ca^2+^/2H^+^ antiport was then explored using LUVs containing the pH-sensitive fluorophore 8-hydroxypyrene-1,3,6-trisulfonic acid (HPTS), in 100 mM CaCl_2_ solution buffered to pH 7 using 10 mM HEPES. A pH gradient was generated by the addition of a 5 mM pulse of NaOH, and the dissipation of the pH gradient by 2a (5 µM) was monitored *via* the ratiometric fluorescence changes of HPTS (*λ*_ex_ = 405/465 nm, *λ*_em_ = 510 nm). The observed transport activity was independent of the presence of FCCP, revealing that 2a mediates Ca^2+^/2H^+^ antiport, with Ca^2+^ transport being rate limiting (Fig. S18). Minimal activity was observed in similar HPTS experiments with 2a (2.5 µM) with Li^+^, Na^+^, K^+^ and Mg^2+^. At higher loading (5 µM), some (comparable) transport for these cations was observed, but reduced with respect to calcium (Fig. S19). Fura-2 experiments replacing the 100 µM Ca^2+^ gradient with Zn^2+^ revealed ∼2-fold enhancement in transport rates, indicating that 2a compares favorably with existing calcium ionophores such as calcimycin that show poor selectivity with respect to competing Zn^2+^ transport (Fig. S11).^35,41^

The Fura-2 assay was also performed in buffered 100 mM NaGluconate solution, where gluconate serves as a large hydrophilic anion that is typically not transported by ionophores. The activity of 2a was unaffected by substituting chloride to gluconate, which rules out the potential Ca^2+^/2Cl^−^ symport mechanism, as well as confirming a lack of non-specific membrane disruption caused by the ionophore (Fig. S12). The rate of transport was substantially reduced when repeating the assay in POPC LUVs containing 30% cholesterol, which lowers the membrane fluidity. This behaviour is characteristic of a mobile carrier mechanism, the rate of which depends on membrane fluidity, in contrast to that typically observed for channel-type mechanisms (Fig. S13).

Together, these results suggest that the observed calcium transport in the Fura-2 assay operates predominantly *via* Ca^2+^/2H^+^ antiport carrier mechanism, with Ca^2+^ transport being rate-limiting under the conditions of the assay, and with minimal contribution from transport of the alkali metal cations *via* Ca^2+^/2M^+^ antiport. This is in agreement with previous observations for the mechanism of Zn^2+^ transport with similar hydroxyquinoline ion carriers.^14^

### Stimuli-responsive ion transport experiments and inter-vesicle signalling

**Fig. 3:**
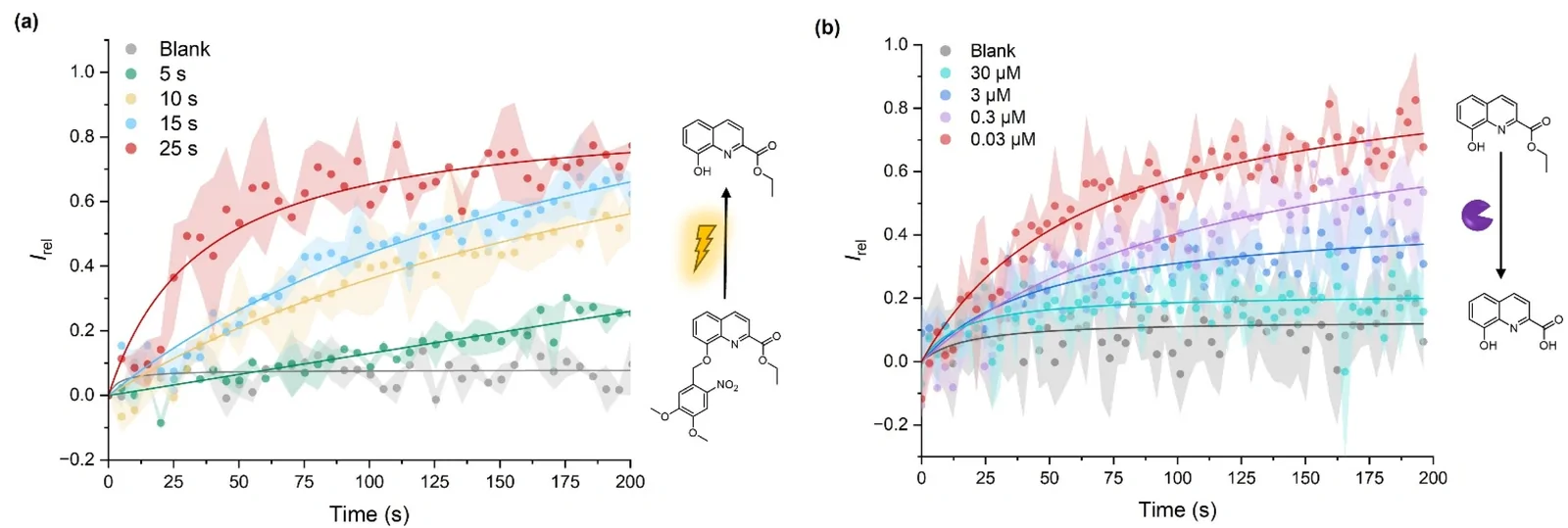
(a) Fura-2 calcium transport assay data for 4 (2.5 µM), following irradiation with 365 nm light (1.3 W LED) for the specified time prior to the start of the assay; (b) Fura-2 calcium transport assay data for 4 (2.5 µM) irradiated at 365 nm for 25 s and following enzyme hydrolysis at the concentrations specified. Assay conditions as in Fig. 2b.

Using light as a stimulus to activate ion transport is an attractive approach to allow for control over the transport process, as it has the advantage of being precise and non-invasive.^46^ Adding a photo-labile protecting group (PPG) onto 2a*via* the phenol oxygen atom enabled photo-responsive behaviour to be integrated into the calcium ionophore, by suppressing transport through obstruction of the cation binding site until the cage is removed with light to reinstate cation binding and hence transport.^47^ This was achieved by functionalising 2a with a 4,5-dimethoxy-2-nitrobenzyl PPG to generate the pro-ionophore 4 (an ‘OFF’ state, Fig. 1a). With the knowledge that compound 1 is inactive for Ca^2+^ transport, a second stimuli-responsive event was designed in which an esterase was used to cleave the ethyl ester on 2a to generate 1, corresponding to a final ‘OFF’ state. The activity of 4 was determined in the Fura-2 Ca^2+^ transport assay (Fig. 3a, grey data). Comparison with 1 and 2a (Fig. 2b) revealed that the PPG in 4 effectively inhibits transport. Thus, both 1 and 4 form transport-inactive ‘OFF’ states, whereas 2a acts as an ‘ON’ state. UV-vis Ca^2+^ binding titration experiments with 4 confirmed that the addition of the PPG fully inhibits binding (Fig. S7).

The ability to generate ionophore 2a from 4 was first investigated by ^1^H NMR experiments. Photo-irradiating a 1 mM solution of 4 at 365 nm with an LED (1.3 W) cleaved the PPG to afford 2a (Fig. S1–S3). Irradiation of a 1 mM sample of 4 in DMSO followed by analysis of Ca^2+^ transport activity using Fura-2-containing LUVs revealed photo-activation of ion transport, the activity of which increased with irradiation time (Fig. 3a). Full activity restoration, comparable to that achieved by a sample of 2a, was reached after 25 s of irradiation. Subsequently incubating this photo-deprotected sample in buffer solution containing 3.0 µM porcine liver esterase for 5 s to hydrolyse 2a and generate 1, followed by addition of the Fura-2 LUVs to initiate the transport assay, revealed that the esterase deactivated transport, therefore generating the second ‘OFF’ state (Fig. S4 and S14). Addition of the esterase to LUVs containing 2a, generated by *ex situ* photo-irradiation of 4, also led to deactivation of transport, the extent of which was correlated to enzyme concentration (Fig. 3b and S16), demonstrating the ability to enzymatically modulate activity in the presence of LUVs.

Diffusible signalling molecules underpin intercellular communication, enabling coordinated behaviour across cell populations.^48^ We and others have developed transmembrane signalling systems that transmit chemical information across membranes without direct mass transport.^49–54^ However, synthetic systems capable of mediating inter-cellular signalling remain exceedingly rare,^55–58^ with most examples relying on biological components for transduction.^59–64^ We have previously shown that a photo-caged hydroxyquinoline zinc ionophore can facilitate inter-vesicle signaling by exploiting a hydrophobic PPG to enable retention within a given vesicle until photo-irradiation releases the ionophore. This then diffuses through the aqueous solution to different vesicles where it facilitates ion transport.^14^ We envisaged exploiting a similar strategy here in which we utilize the photo- and enzyme-responsive calcium ionophores to mediate controllable inter-vesicle communication.

**Fig. 4:**
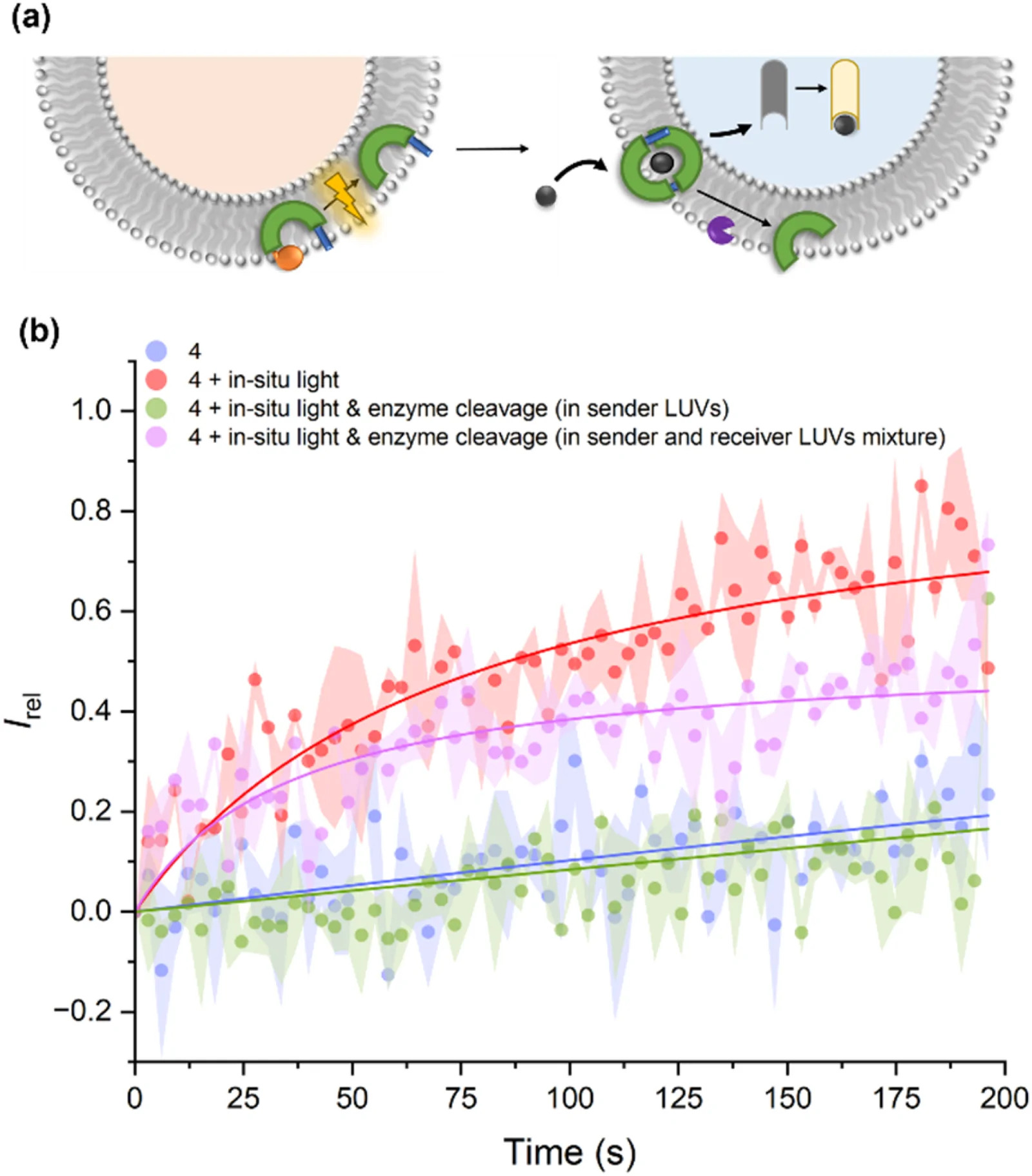
. (a) Schematic of photo- and enzyme-regulated inter-vesicle signalling process between sender (orange) and receiver (blue) vesicles, triggering and deactivating calcium influx into the receiver LUVs respectively; (b) Fura-2 calcium transport assay data in POPC receiver vesicles for 4 (2.5 µM) irradiated at 365 nm for 150 s in the sender LUVs (red data), and without irradiation (blue data). Addition of porcine liver esterase (30 µM) to photo-irradiated 4 in sender LUVs (prior to the start of the assay; green data) or in the presence of both sender and receiver LUVs (during the assay run; purple data) deactivates calcium influx in the receiver vesicles.

We designed an experiment in which the ionophore acts as an inter-vesicle signal, triggering calcium influx into a remote population of vesicle following its release from a different population. To achieve this, two populations of distinct POPC LUVs were prepared: the sender vesicles which were vacant, containing only the aqueous buffer solution inside and hydrophobic pro-ionophore 4 encapsulated in the membrane; and the receiver vesicles which contained Fura-2 to detect calcium influx. The sender vesicles were photo-irradiated for various time lengths, after which the receiver LUVs were introduced in the presence of 100 µM CaCl_2_ in the external solution. Ca^2+^ influx into the receiver vesicles was monitored over time *via* the changing ratiometric fluorescence of Fura-2. No change in fluorescence was observed when the sender and receiver vesicles were mixed in the absence of photo-irradiation (Fig. 4b, blue data), whilst when the sender vesicles were first irradiated at 365 nm, this initiated calcium transport into the receiver vesicles at a rate comparable to that facilitated by direct addition of 2a (Fig. 4b, red data and S17). This is indicative of near quantitative release of 2a from the sender vesicles, and subsequent uptake into the receiver vesicles where they facilitate calcium influx.

Finally, calcium influx in the receiver vesicles could be deactivated in the presence of the esterase enzyme. Incubation of 2a in sender vesicles with enzyme to generate 1 prior to the start of the experiment completely inhibited signalling and no calcium influx into the receiver vesicles was observed (Fig. 4b, green data). Addition of enzyme to the LUV suspension at the start of the signalling experiment enabled regulation of calcium influx in real-time, by deactivating 2a*in situ* (Fig. 4b, purple data). Overall, these experiments demonstrate the ability of 2a to diffuse between vesicles and enable inter-vesicle signalling which itself is up- and down-regulated in the presence of light and an enzyme.

## Conclusions

We have developed the first example of a stimuli-responsive calcium ionophore. By integrating photo- and enzyme-responsive moieties to the ionophore, a dual-stimuli responsive OFF–ON–OFF transmembrane transport system derived from a tridentate hydroxyquinoline scaffold was accessed. Calcium influx into vesicles was monitored using complementary fluorescence assays, employing encapsulated Fura-2 for bulk measurements and Fluo-8 in a single-vesicle confocal assay. The responsive ionophore was applied to engineer an inter-vesicle signalling system, in which activation of calcium influx in a remote population of vesicles could be up and down regulated by light and enzymes, respectively. This approach recapitulates some key features of biological signalling, employing diffusible chemical signals to coordinate activity between compartments. We anticipate that these results may lead to the development of increasingly sophisticated intercellular communication networks, enabling direct information exchange between artificial and living cellular systems. Furthermore, it opens new avenues for integrating small-molecule, photo-responsive ionophores into optogenetic platforms, providing a means for precise, light-controlled regulation of chemical communication.

## Author contributions

G. K. performed the synthesis and characterisation of the compounds, the Fura-2, HPTS and ISE transport assay studies. K. M. B. and D. C. E. performed the single-liposome assay studies. M. H. H. and S. L. C. supervised the single-liposome component of the work. G. K. wrote the first draft of the manuscript, which was edited by all authors. M. J. L. supervised the project.

## Conflicts of interest

There are no conflicts to declare.

## Supplementary Material

SC-017-D6SC02506D-s001

## Data Availability

All data supporting the findings of this study are available within the article and the supplementary information (SI). Supplementary information is available. See DOI: https://doi.org/10.1039/d6sc02506d.

## References

[cit1] Langton M. J. (2021). Nat. Rev. Chem..

[cit2] Davis J. T., Okunola O., Quesada R. (2010). Chem. Soc. Rev..

[cit3] Matile S., Vargas Jentzsch A., Montenegro J., Fin A. (2011). Chem. Soc. Rev..

[cit4] Yang J., Yu G., Sessler J. L., Shin I., Gale P. A., Huang F. (2021). Chem.

[cit5] Davis J. T., Gale P. A., Quesada R. (2020). Chem. Soc. Rev..

[cit6] Bickerton L. E., Johnson T. G., Kerckhoffs A., Langton M. J. (2021). Chem. Sci..

[cit7] Steinbrueck A., Sedgwick A. C., Brewster J. T., Yan K.-C., Shang Y., Knoll D. M., Vargas-Zúñiga G. I., He X.-P., Tian H., Sessler J. L. (2020). Chem. Soc. Rev..

[cit8] Tardito S., Bassanetti I., Bignardi C., Elviri L., Tegoni M., Mucchino C., Bussolati O., Franchi-Gazzola R., Marchiò L. (2011). J. Am. Chem. Soc..

[cit9] Magda D., Lecane P., Wang Z., Hu W., Thiemann P., Ma X., Dranchak P. K., Wang X., Lynch V., Wei W., Csokai V., Hacia J. G., Sessler J. L. (2008). Cancer Res..

[cit10] Choi Y. R., Kim G. C., Jeon H.-G., Park J., Namkung W., Jeong K.-S. (2014). Chem. Commun..

[cit11] Ahmad M., Metya S., Das A., Talukdar P. (2020). Chem. Eur. J..

[cit12] Kerckhoffs A., Langton M. J. (2020). Chem. Sci..

[cit13] Wezenberg S. J., Chen L.-J., Bos J. E., Feringa B. L., Howe E. N. W., Wu X., Siegler M. A., Gale P. A. (2022). J. Am. Chem. Soc..

[cit14] Gartland S. A., Johnson T. G., Walkley E., Langton M. J. (2023). Angew. Chem., Int. Ed..

[cit15] Chao X., Johnson T. G., Temian M.-C., Docker A., Wallabregue A. L. D., Scott A., Conway S. J., Langton M. J. (2024). J. Am. Chem. Soc..

[cit16] Zawada B., Chmielewski M. J. (2024). Org. Biomol. Chem..

[cit17] Chattopadhayay S., Wanjari P., Talukdar P. (2024). Chem. Sci..

[cit18] Grählert E., Langton M. J. (2025). Angew. Chem., Int. Ed..

[cit19] Docker A., Johnson T. G., Kuhn H., Zhang Z., Langton M. J. (2023). J. Am. Chem. Soc..

[cit20] Park G., Gabbaï F. P. (2020). Chem. Sci..

[cit21] Fares M., Wu X., Ramesh D., Lewis W., Keller P. A., Howe E. N. W., Pérez-Tomás R., Gale P. A. (2020). Angew. Chem., Int. Ed..

[cit22] Choi Y. R., Lee B., Park J., Namkung W., Jeong K.-S. (2016). J. Am. Chem. Soc..

[cit23] Ahmad M., Johnson T. G., Flerin M., Duarte F., Langton M. J. (2024). Angew. Chem., Int. Ed..

[cit24] Simms B. A., Zamponi G. W. (2014). Neuron.

[cit25] Clapham D. E. (2007). Cell.

[cit26] Berridge M. J., Lipp P., Bootman M. D. (2000). Nat. Rev..

[cit27] Orrenius S., Zhivotovsky B., Nicotera P. (2003). Nat. Rev. Mol. Cell Biol..

[cit28] Cain S. M., Snutch T. P. (2011). BioFactors.

[cit29] Saha P., Basak D., Biswas S., More P. A., Madhavan N. (2022). Bioconjug. Chem..

[cit30] Lin Z., Yu S., Chen J., Tian J., Xu Z., Yao C., Dong Z. (2025). Chin. J. Chem..

[cit31] Elie C.-R., Hébert A., Charbonneau M., Haiun A., Schmitzer A. R. (2013). Org. Biomol. Chem..

[cit32] Kawano R., Horike N., Hijikata Y., Kondo M., Carné-Sánchez A., Larpent P., Ikemura S., Osaki T., Kamiya K., Kitagawa S., Takeuchi S., Furukawa S. (2017). Chem.

[cit33] Liu C., Hermann T. E. (1978). J. Biol. Chem..

[cit34] Hu T. Q., Weiler L. (1994). Can. J. Chem..

[cit35] Erdahl W. L., Chapman C. J., Wang E., Taylor R. W., Pfeiffer D. R. (1996). Biochem..

[cit36] Wang E., Erdahl W. L., Hamidinia S. A., Chapman C. J., Taylor R. W., Pfeiffer D. R. (2001). Biophys. J..

[cit37] Fernandez Lahore R. G., Pampaloni N. P., Schiewer E., Heim M. M., Tillert L., Vierock J., Oppermann J., Walther J., Schmitz D., Owald D., Plested A. J. R., Rost B. R., Hegemann P. (2022). Nat. Commun..

[cit38] Piatkevich K. D., Boyden E. S. (2023). Q. Rev. Biophys..

[cit39] Deisseroth K. (2011). Nat. Methods.

[cit40] Miesenböck G. (2011). Annu. Rev. Cell Dev. Biol..

[cit41] Reed P. W., Lardy H. A. (1972). J. Biol. Chem..

[cit42] Berendes R., Burger A., Voges D., Demange P., Huber R. (1993). FEBS Lett..

[cit43] Grynkiewicz G., Poenie M., Tsien R. Y. (1985). J.
Biol. Chem..

[cit44] Busschaert N., Bradberry S. J., Wenzel M., Haynes C. J. E., Hiscock J. R., Kirby I. L., Karagiannidis L. E., Moore S. J., Wells N. J., Herniman J., Langley G. J., Horton P. N., Light M. E., Marques I., Costa P. J., Félix V., Frey J. G., Gale P. A. (2013). Chem. Sci..

[cit45] Bąk K. M., Edwards D. C., George D., Singh B., Ferguson R., Zhao T., Piché K., Louwrier A., Cockroft S. L., Horrocks M. H. (2025). Angew. Chem., Int. Ed..

[cit46] Velema W. A., Szymanski W., Feringa B. L. (2014). J. Am. Chem. Soc..

[cit47] Ellis-Davies G. C. R. (2007). Nat. Methods.

[cit48] Krauss G. , Biochemistry of Signal Transduction and Regulation, Wiley, New York, 2006

[cit49] Bernitzki K., Maue M., Schrader T. (2012). Chem. Eur. J..

[cit50] Lister F. G. A., Le Bailly B. A. F., Webb S. J., Clayden J. (2017). Nat. Chem..

[cit51] Barton P., Hunter C. A., Potter T. J., Webb S. J., Williams N. H. (2002). Angew. Chem., Int. Ed..

[cit52] Langton M. J., Scriven L. M., Williams N. H., Hunter C. A. (2017). J. Am. Chem. Soc..

[cit53] Langton M. J., Williams N. H., Hunter C. A. (2017). J. Am. Chem. Soc..

[cit54] Langton M. J., Keymeulen F., Ciaccia M., Williams N. H., Hunter C. A. (2017). Nat. Chem..

[cit55] Ding Y., Williams N. H., Hunter C. A. (2019). J. Am. Chem. Soc..

[cit56] Bernitzki K., Schrader T. (2009). Angew. Chem., Int. Ed..

[cit57] Bekus R., Rudolph K., Riebe S., Voskuhl J., Schrader T. (2025). ChemPlusChem.

[cit58] Søgaard A. B., Pedersen A. B., Løvschall K. B., Monge P., Jakobsen J. H., Džabbarova L., Nielsen L. F., Stevanovic S., Walther R., Zelikin A. N. (2023). Nat. Commun..

[cit59] Gardner P. M., Winzer K., Davis B. G. (2009). Nat. Chem..

[cit60] Lentini R., Santero S. P., Chizzolini F., Cecchi D., Fontana J., Marchioretto M., Del Bianco C., Terrell J. L., Spencer A. C., Martini L., Forlin M., Assfalg M., Serra M. D., Bentley W. E., Mansy S. S. (2014). Nat. Commun..

[cit61] De Luis B., Llopis-Lorente A., Sancenón F., Martínez-Máñez R. (2021). Chem. Soc. Rev..

[cit62] Llopis-Lorente A., Díez P., Sánchez A., Marcos M. D., Sancenón F., Martínez-Ruiz P., Villalonga R., Martínez-Máñez R. (2017). Nat. Commun..

[cit63] Adamala K. P., Martin-Alarcon D. A., Guthrie-Honea K. R., Boyden E. S. (2017). Nat. Chem..

[cit64] Buddingh’ B. C., Elzinga J., Van Hest J. C. M. (2020). Nat. Commun..

