# Supplementary material for: Dual-stimuli responsive ionophore for OFF–ON–OFF transmembrane calcium ion transport and inter-vesicle signalling
Source: Chem Sci. 2026 Jul 1;17(33):15868–74. doi: 10.1039/d6sc02506d (PMC13344878; doi:10.1039/d6sc02506d)
Supplement: Supplementary file 1 [file SC-017-D6SC02506D-s001.pdf]

Supplementary Information (SI) for Chemical Science.
This journal is © The Royal Society of Chemistry 2026

Supporting Information

Dual-Stimuli Responsive Ionophore for OFF- ON- OFF Transmembrane Calcium Ion Transport and Inter-vesicle Signalling

Gurshinder Kaur,[a] Krzysztof M. Bąk,[b] Daniel C. Edwards,[b] Scott L. Cockroft,[b] Mathew H. Horrocks,[bc] Matthew J. Langton*[a]

[a] Chemistry Research Laboratory, University of Oxford, Mansfield Road, Oxford, OX1 3TA, UK
[b] EaStCHEM School of Chemistry, University of Edinburgh, Joseph Black Building, David Brewster Rd, Edinburgh, EH9 3FJ, UK
[c] IRR Chemistry Hub, Institute of Regeneration and Repair, University of Edinburgh, Edinburgh, EH16 4UU, UK

Content

1. Abbreviations 2
2. Materials and Methods.. 3
3. Synthesis and Characterisation 4
4. ^{1}H-NMR Photo-decaging Experiment 12
5. Mass Spectrometry Data 14
6. UV-vis Binding Titrations 15
7. Fura-2 Assay Ca^{2+} Transport Experiments 17
8. HPTS Assay Studies 25
9. Calcium Ion Selective Electrode (ISE) Assay Experiments 27
10. Single Liposome Calcium Transport Assay 30
11. References.. 33

1. Abbreviations

DMSO Dimethyl sulfoxide

DPPC Dipalmitoylphosphatidylcholine

EDC 1-Ethyl-3-(3-dimethylaminopropyl)carbodiimide

EDTA Ethylenediaminetetraacetic acid

FCCP Carbonyl cyanide-*p*-trifluoromethoxyphenylhydrazone

HEPES 2-[4-(2-hydroxyethyl)piperazin-1-yl]ethanesulfonic acid

HPTS 8-hydroxypyrene-1,3,6-trisulfonic acid

HRMS High resolution mass spectrometry

ISE Ion selective electrode

LUVs Large unilamellar vesicles

NMR Nuclear magnetic resonance

POPC 1-Palmitoyl-2-oleoyl-glycero-3-phosphocholine

PPG Photoremovable protecting group

ppm Parts per million

rt Room temperature

TLC Thin layer chromatography

UV-vis Ultraviolet-visible

w.r.t. With respect to

2. Materials and Methods

All reagents and solvents were acquired from various commercial suppliers and used as purchased unless stated otherwise. Lipids were purchased from Avanti polar lipids and used without further purification. Esterase from porcine liver was purchased from Sigma Aldrich. Where necessary, solvents were dried by passing through an MBraun MPSP-800 column and degassed with nitrogen. Normal phase silica gel flash column chromatography was performed manually using Merck® silica gel 60 under a positive pressure of nitrogen. Solvent mixtures are reported by volume where indicated. For TLC monitoring, Merck silica 60 F254 plates with UV fluorescence (254 nm) were used. NMR spectra were recorded on Bruker AVIII HD 400, Bruker NEO 600 with broadband helium cryoprobe, and Bruker AVIII 600 spectrometers. All chemical shifts are reported as δ values in ppm. ^{1}H-NMR signal splitting patterns are abbreviated as follows: s (singlet), d (doublet), dd (doublet of doublet), ddd (doublet of doublet of doublet), t (triplet), td (triplet of doublets), m (multiplet), brs (broad singlet) and br q (broad quartet). MS spectra were acquired on a Waters BioAccord LC-MS system; flow injection analysis was performed on an ACQUITY I-Class PLUS UPLC System (Waters, Millford, MA, USA) coupled to an AQUITY RDa mass spectrometer (Waters, Milford, MA, USA) equipped with an ESI probe, in positive ion mode. Fluorescence spectroscopic data were recorded using a Horiba Duetta fluorescence spectrophotometer, equipped with a Peltier temperature controller and stirrer. Experiments were conducted at 25 °C, unless otherwise stated. Vesicles were prepared as described below using Avestin "LiposoFast" extruder apparatus, equipped with polycarbonate membranes with 200 nm pores. GPC purification of vesicles was carried out using GE Healthcare PD-10 desalting columns prepacked with Sephadex G 25 medium. Photo-irradiation of liquid samples was carried out using Thorlabs high-power mounted LEDs (M365L2- 365 m, 190 mW). Calcium standard solution (0.1 M) for the calibration of the calcium selective electrode (Thermo Scientific™ Orion™ Sure-Flow® Combination Calcium Electrode ISE) was purchased from Fisher Scientific. Calculated log P values (clogP) were determined using the VCClab software.[1]

3. Synthesis and Characterisation

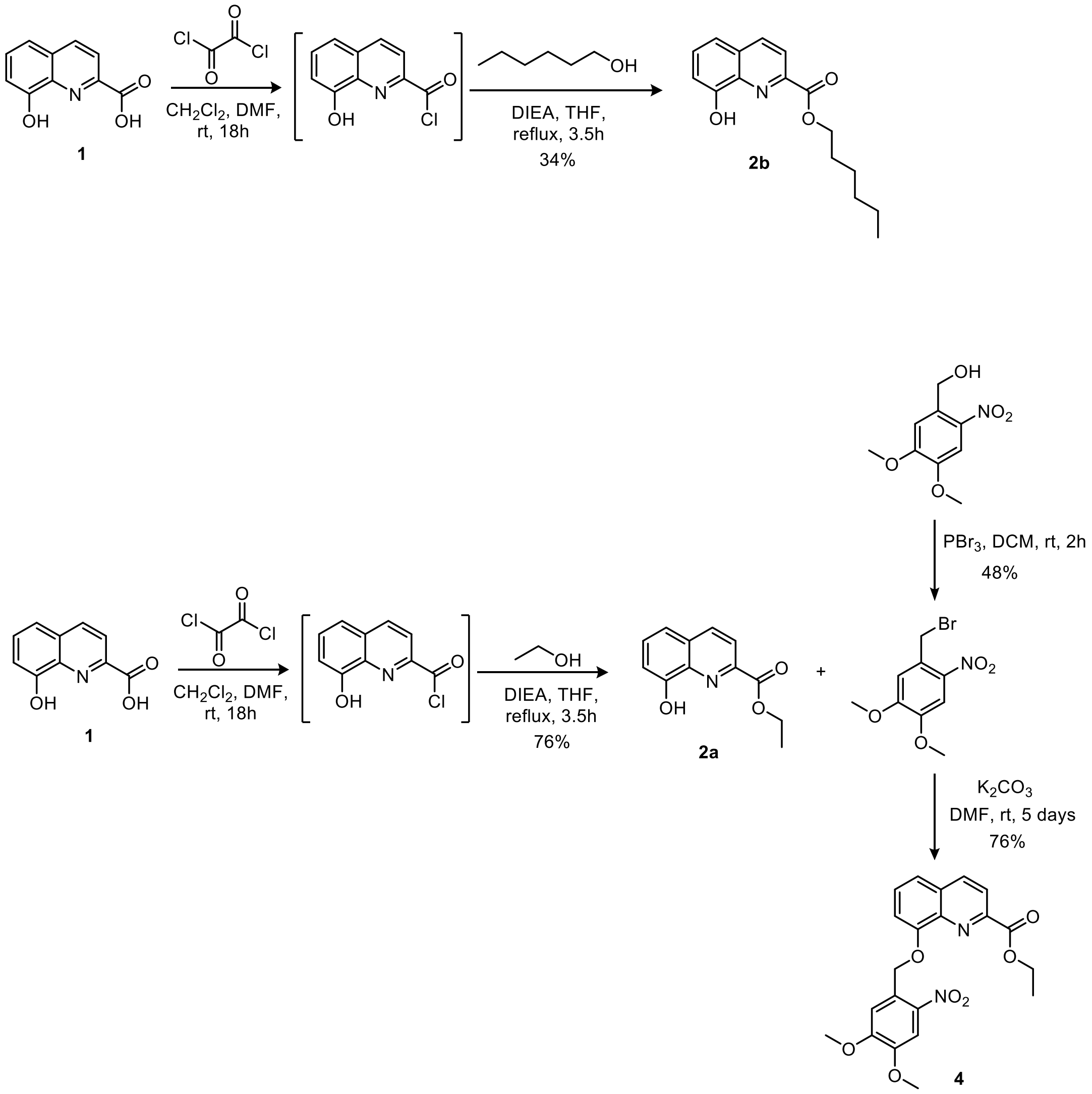

Scheme S1: synthesis of compound **2a**, **2b** and **4**, starting from commercially available compound **1**.

Synthesis of compound **2b**

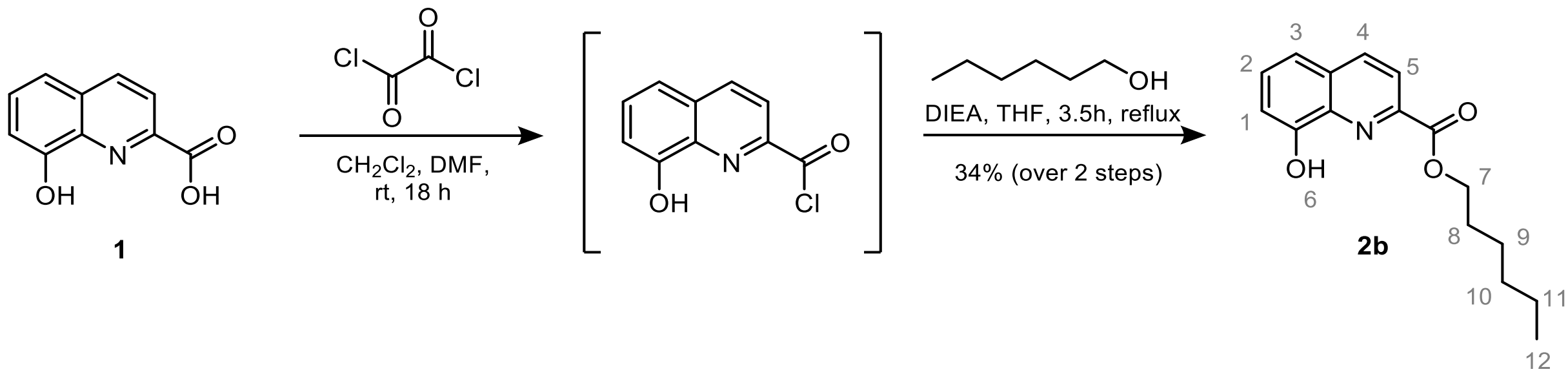

To a suspension of commercially available 8-hydroxyquinoline-2-carboxylic acid (102 mg, 537 μmol, 1.00 eq.) in anhydrous CH_2Cl_2 (11 mL), oxalyl chloride (0.30 mL, 3.49 mmol, 6.5 eq.) and anhydrous DMF (0.1 mL) were added. The mixture was stirred at room temperature for 18 hours. The solvent was removed *in vacuo* and the product was dried for 90 minutes on high vacuum. To this solid, hexanol (0.10 mL, 805 μmol, 1.5 eq.), DIEA (0.11 mL, 628 μmol, 1.17 eq.) and anhydrous THF (4.00 mL) were added. The resulting mixture was refluxed for 3.5 hours. The mixture was washed with water (50 mL) and extracted with EtOAc (50 mL). The organic layer was dried over $MgSO_4$ and the solvent was removed *in vacuo*. The crude was purified by silica gel flash chromatography (10% EtOAc in hexane) to afford the product as a yellow solid (49.5 mg, 181 μmol, 34% over two steps). ^{1}H-NMR (600 MHz, $CDCl_3$) δ 8.56 (brs, 1H, O*H*), 8.27- 8.25 (d, *J*= 12 Hz, 1H, Ar*H*), 8.15- 8.14 (d, *J*= 6 Hz, 1H, Ar*H*), 7.56- 7.53 (t, *J*= 18 Hz, 6 Hz, 1H, Ar*H*), 7.37- 7.36 (dd, *J*= 6 Hz, 1H, Ar*H*), 7.23- 7.22 (dd, *J*= 6 Hz, 1H, Ar*H*), 4.45- 4.43 (t, *J*= 12 Hz, 6Hz, 2H, C*H₂*), 1.86- 1.81 (m, 2H, C*H₂*), 1.50- 1.45 (m, 2H, C*H₂*), 1.39- 1.32 (m, 4H, C*H₂*C*H₂*), 0.92- 0.89 (m, 3H, C*H₃*). ^{13}C-NMR (151 MHz, $CDCl_3$) δ 165.2, 153.4, 145.8, 137.9, 137.3, 130.3, 129.8, 121.7, 117.7, 111.1, 66.4, 31.6, 28.7, 25.8, 22.7, 14.1. HMRS (ES+) Calc. for $C_{16}H_{20}NO_3$ $[M+H]^+$ = 274.1438, found $[M+H]^+$ = 274.1448.

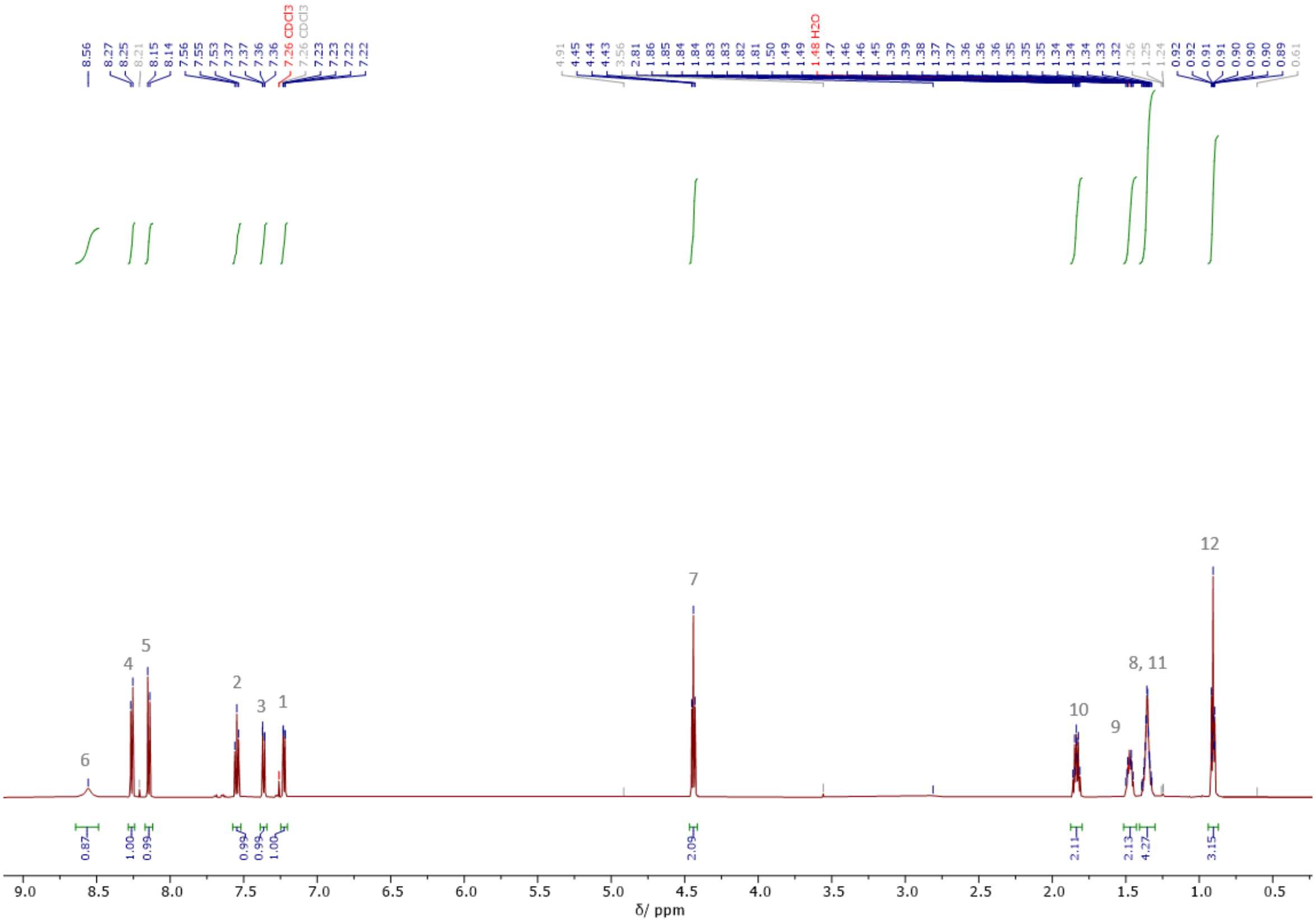

^{1}H-NMR (600 MHz, $CDCl_3$, 298 K) of compound **2b**

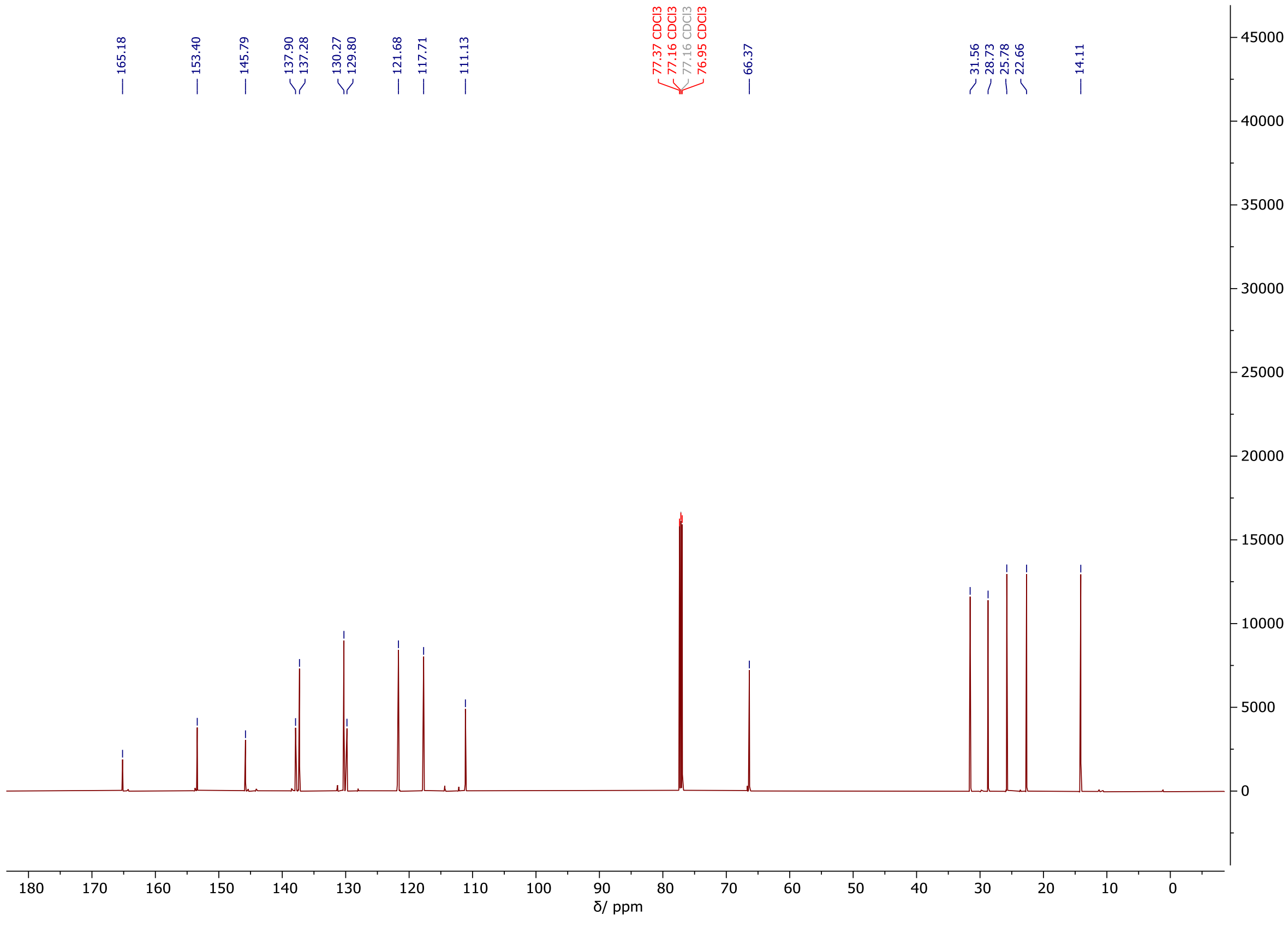

^{13}C-NMR (151 MHz, $CDCl_3$, 298 K) of compound **2b**

Synthesis of compound **2a**

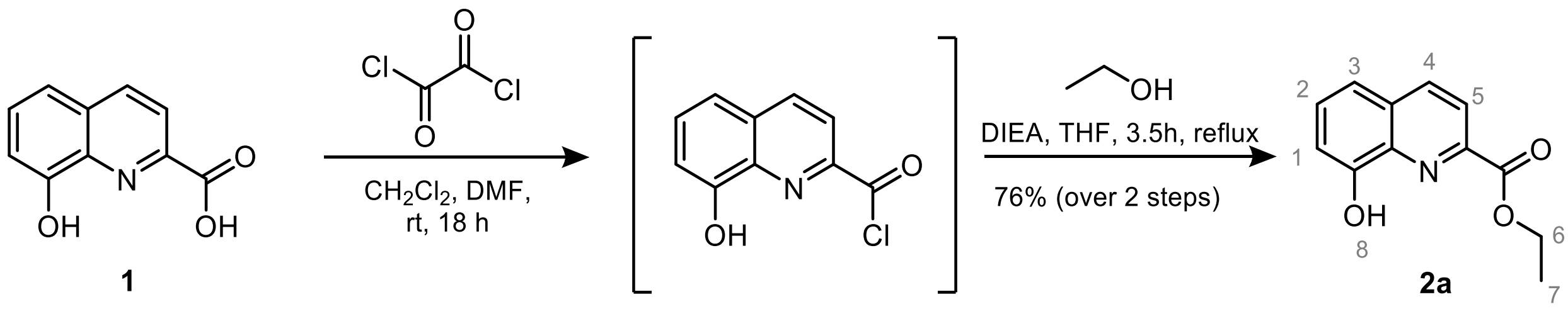

To a suspension of commercially available 8-hydroxyquinoline-2-carboxylic acid (101 mg, 531 µmol, 1.00 eq.) in anhydrous CH_2Cl_2 (11 mL), oxalyl chloride (0.23 mL, mmol, 5.1 eq.) and few drops of anhydrous DMF were added. The mixture was stirred at room temperature for 18 hours. The solvent was removed *in vacuo* and the product was dried for 90 minutes under high vacuum. To this solid, EtOH (4 mL), DIEA (0.11 mL, 622 µmol, 1.17 eq.) and anhydrous THF (4.00 mL) were added. The resulting mixture was refluxed for 3.5 hours. The mixture was diluted with EtOAc (50 mL) and washed with water (50 mL). The organic layer was dried over $MgSO_4$ and the solvent was removed *in vacuo*. The crude was purified by silica gel flash chromatography (20% EtOAc in hexane) to afford the product as a yellow solid (87.5 mg, 403 µmol, 76% over two steps). ^{1}H-NMR (600 MHz, $CDCl_3$) δ 8.49 (brs, 1H, O*H*), 8.28 (d, *J* = 8.5 Hz, 1H, Ar*H*), 8.17 (d, *J* = 8.5 Hz, 1H, Ar*H*), 7.58 – 7.54 (m, 1H, Ar*H*), 7.38 (dd, *J* = 8.3, 1.2 Hz, 1H, Ar*H*), 7.23 (dd, *J* = 7.6, 1.2 Hz, 1H, Ar*H*), 4.52 (q, *J* = 7.1 Hz, 2H, C*H₂*), 1.48 (t, *J* = 7.2 Hz, 3H, C*H₃*). ^{13}C-NMR (151 MHz, $CDCl_3$) 165.2, 153.4, 145.8, 137.9, 137.3, 130.3, 129.8, 121.8, 117.8, 111.1, 62.2, 14.5. HMRS (ES+) Calc. for $C_{12}H_{12}NO_3$ $[M+H]^+$ = 218.0812, found $[M+H]^+$ = 218.0805.

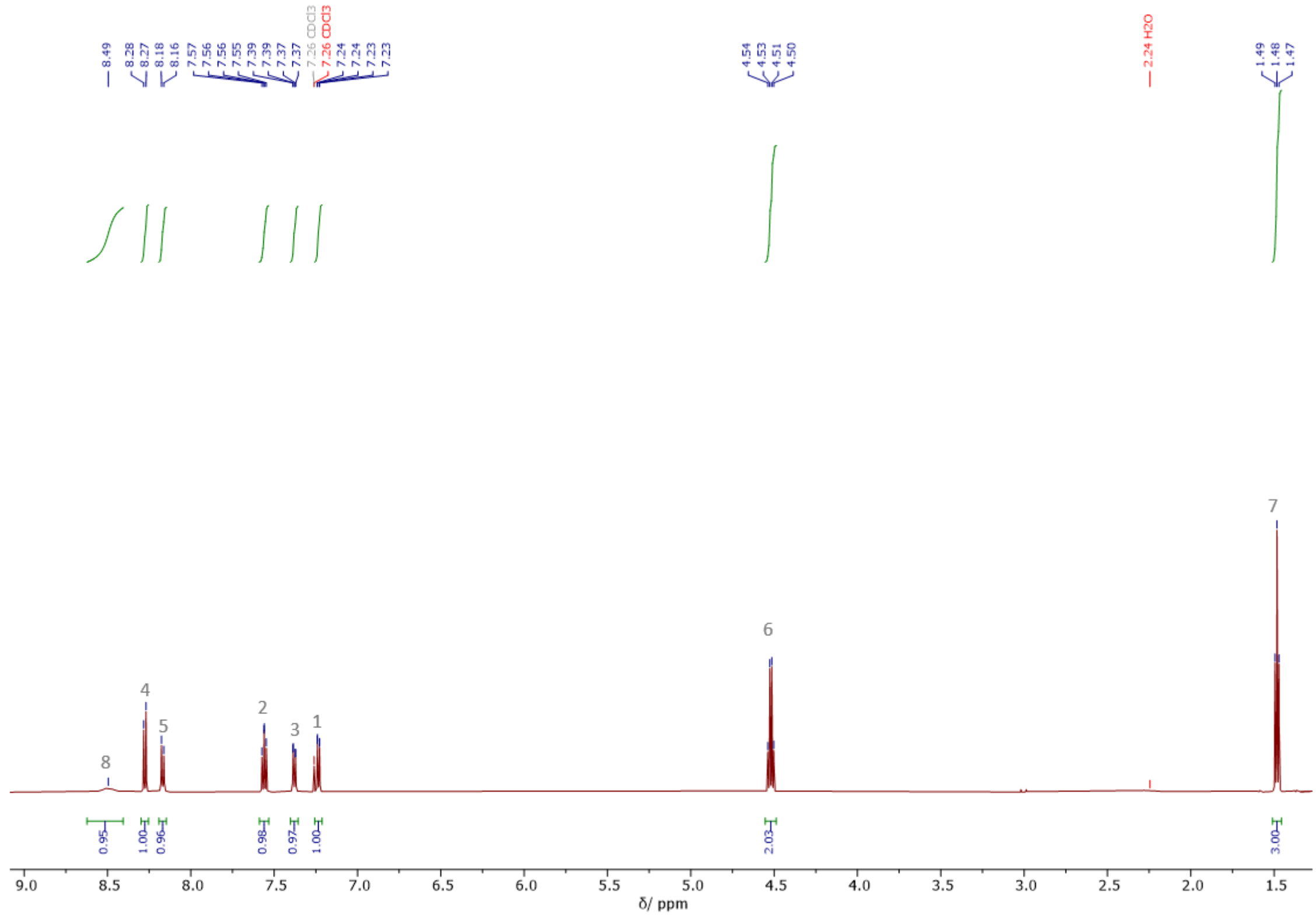

^{1}H-NMR (600 MHz, $CDCl_3$, 298 K) of compound **2a**

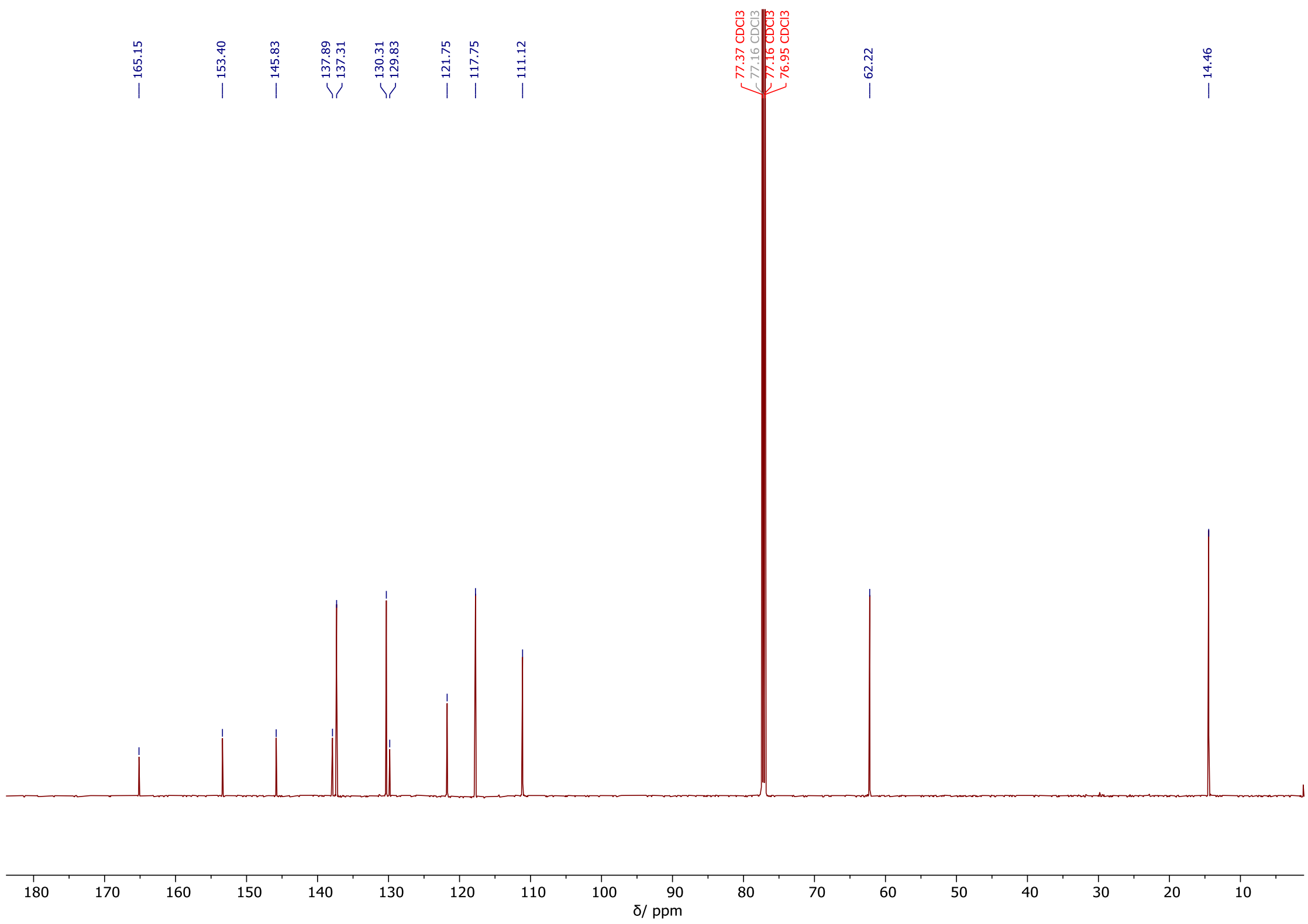

^{13}C-NMR (151 MHz, $CDCl_3$, 298 K) of compound **2a**

Synthesis of the PPG, 4,5-dimethoxy-2-nitrobenzyl bromide

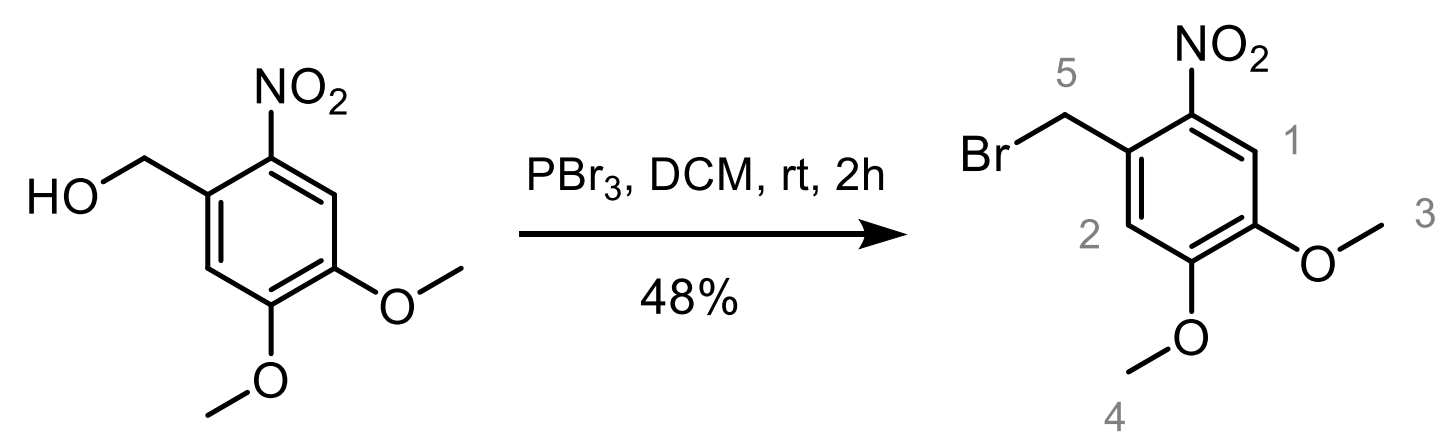

4,5-Dimethoxy-2-nitrophenyl methanol (2.50 g, 11.7 mmol, 1.0 eq.) was dissolved in anhydrous DCM (80 mL). Under external cooling, phosphorus tribromide (1.21 mL, 12.9 mmol, 1.1 eq.) was added dropwise via syringe and the mixture was stirred at room temperature for 2 hours. Reaction was neutralised with saturated $NaHCO_3$ solution (80 mL). The organic layer was extracted, dried over $MgSO_4$ and concentrated in vacuo. The crude was purified by silica gel flash chromatography (20% EtOAc in pentane) to afford the product as an orange solid (1.56 g, 5.65 mmol, 48%). ^{1}H-NMR (400 MHz, $CDCl_3$) δ 7.67 (s, 1H, Ar*H*), 6.94 (s, 1H, Ar*H*), 4.87 (s, 2H, CH_2), 4.00 (s, 3H, CH_3), 3.96 (s, 3H, CH_3). HRMS (ES+) Calc. for $C_9H_{11}BrNO_4$ $[M+H]^+$ = 275.9866, found $[M+H]^+$ = 275.9879, consistent with the literature.[2]

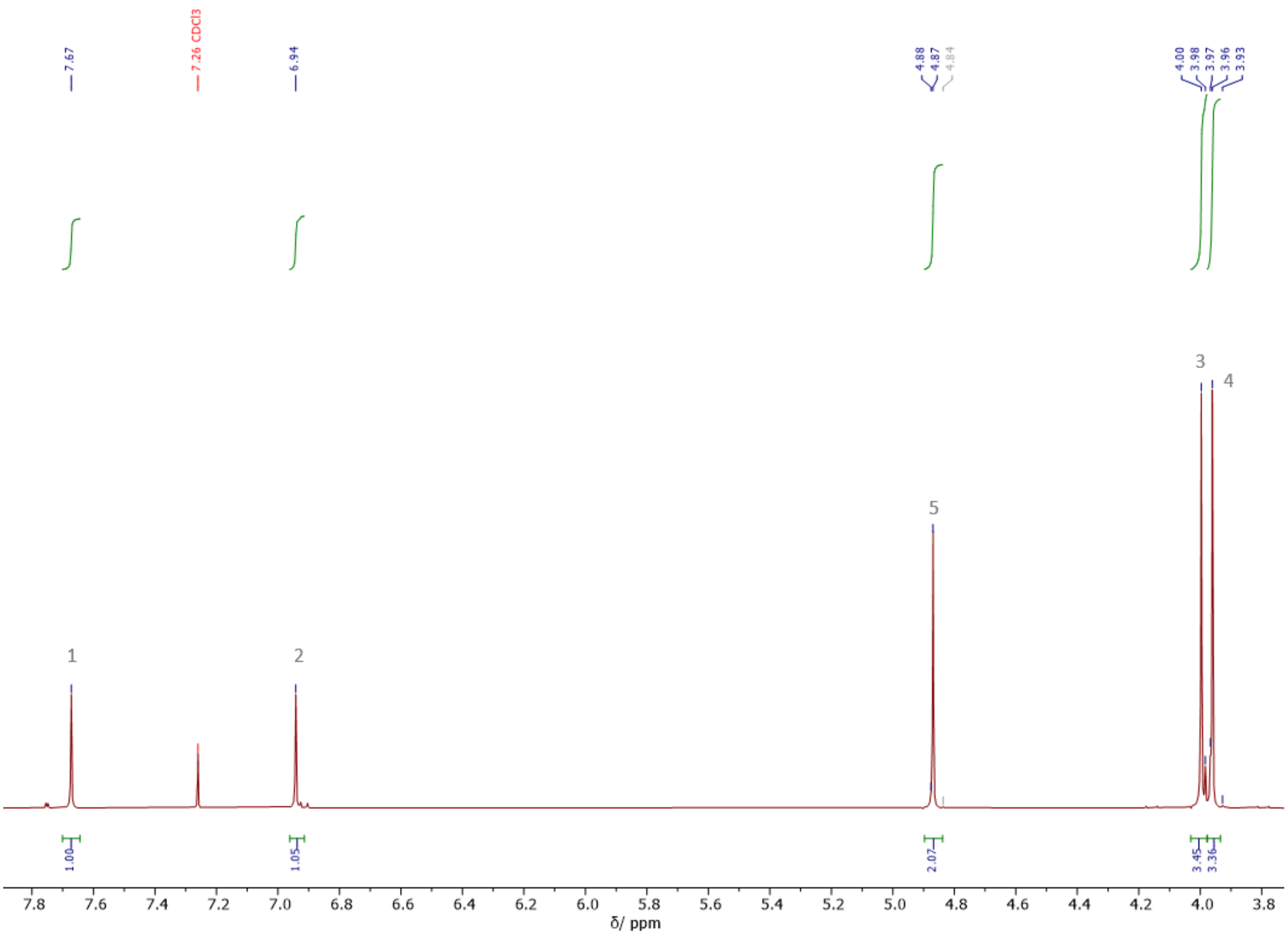

^{1}H-NMR (400 MHz, $CDCl_3$, 298 K) of PPG

Synthesis of compound **4**

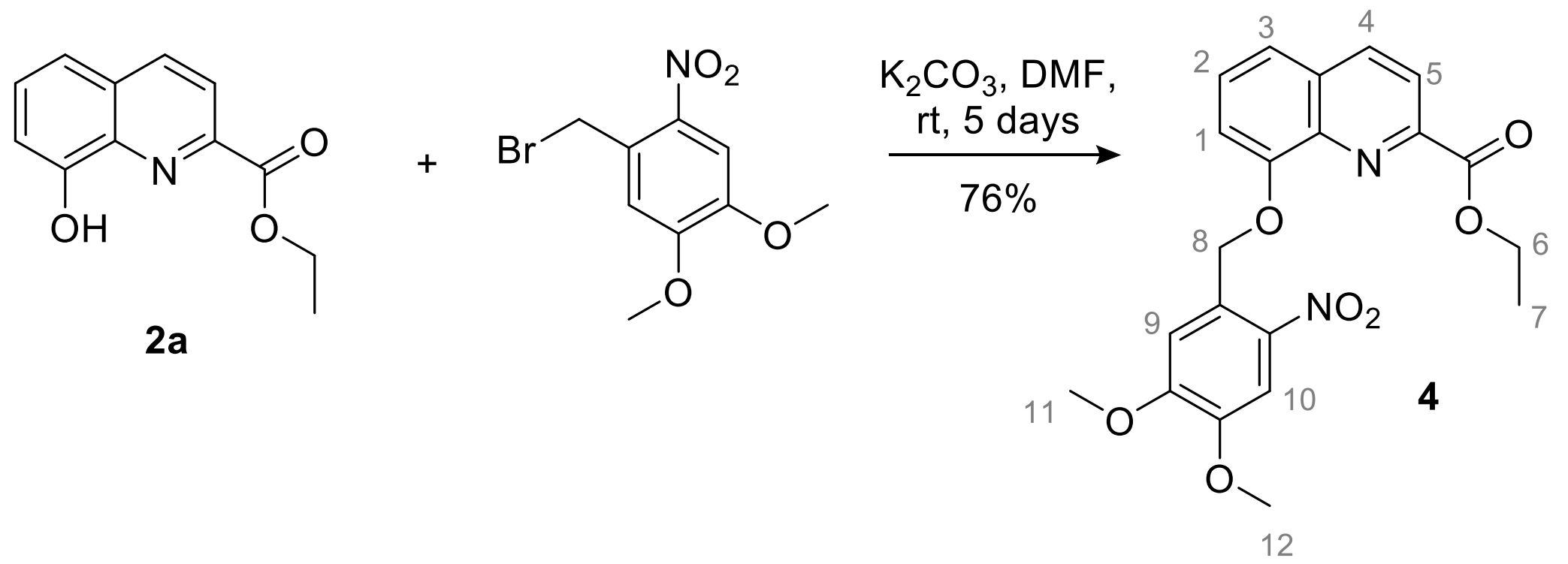

To stirred solution of compound **2a** (26.7 mg, 123 µmol, 1.00 eq.) in anhydrous DMF (2 mL), K_2CO_3 (69.0 mg, mmol, 4.10 eq.) and 4,5-dimethoxy-2-nitrobenzyl bromide (34.4 mg, µmol, 1.01 eq.) were added. Resulting suspension was stirred for 5 days at room temperature. The reaction mixture was diluted with EtOAc (10mL) and washed with 5% LiCl solution (10 mL) and brine (10 mL). The organic layer was dried over $MgSO_4$ and concentrated *in vacuo*. The crude was purified by silica gel flash chromatography (20% EtOAc in hexane) to afford the product as a yellow solid (38.4 mg, 93.1 µmol, 76%). ^{1}H-NMR (600 MHz, $CDCl_3$) δ 8.30 (d, *J* = 8.5 Hz, 1H, Ar*H*), 8.21 (d, *J* = 8.5 Hz, 1H, Ar*H*), 8.11 (s, 1H, Ar*H*), 7.82 (s, 1H, Ar*H*), 7.60 (t, *J* = 8.0 Hz, 1H, Ar*H*), 7.51 (dd, *J* = 8.2, 1.2 Hz, 1H, Ar*H*), 7.31 – 7.27 (m, 1H, Ar*H*), 5.77 (s, 2H, C*H₂*), 4.51 (q, *J* = 7.2 Hz, 2H, C*H₂*), 4.16 (s, 3H, OC*H₃*), 3.99 (s, 3H, OC*H₃*), 1.46 (dd, *J* = 7.6, 6.8 Hz, 3H, CH_3). ^{13}C-NMR (151 MHz, $CDCl_3$) δ 165.3, 154.7, 154.6, 147.9, 147.5, 140.2, 138.7, 137.2, 130.7, 129.9, 129.2, 121.9, 120.2, 110.4, 110.1, 108.0, 67.7, 62.1, 57.0, 56.5, 14.6, 1.2. HRMS (ES+) Calc. for $C_{21}H_{20}N_2O_7Na$ $[M+Na]^+$ = 435.1163, found $[M+Na]^+$ = 435.1181.

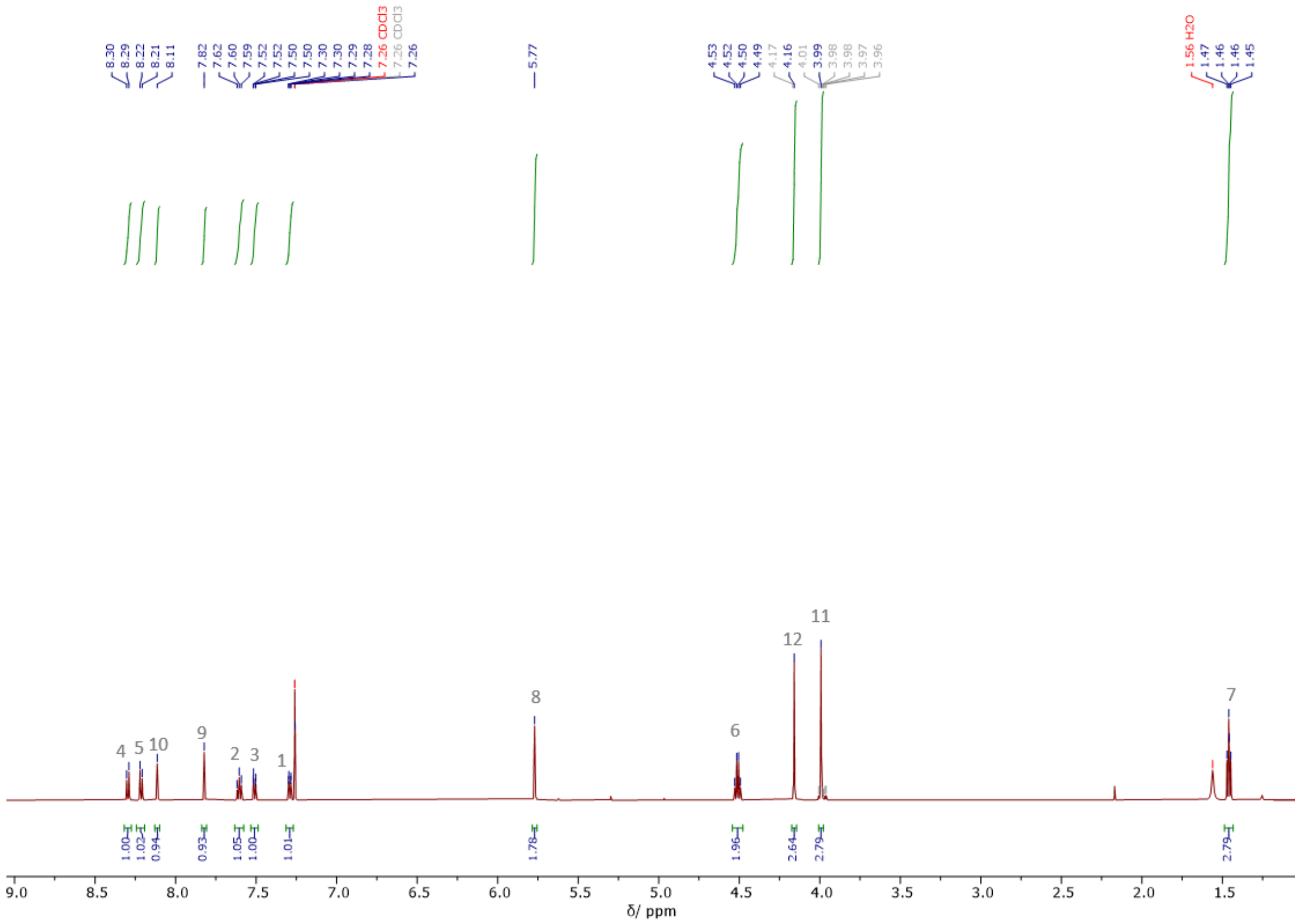

^{1}H-NMR (600 MHz, $CDCl_3$, 298 K) of compound **4**

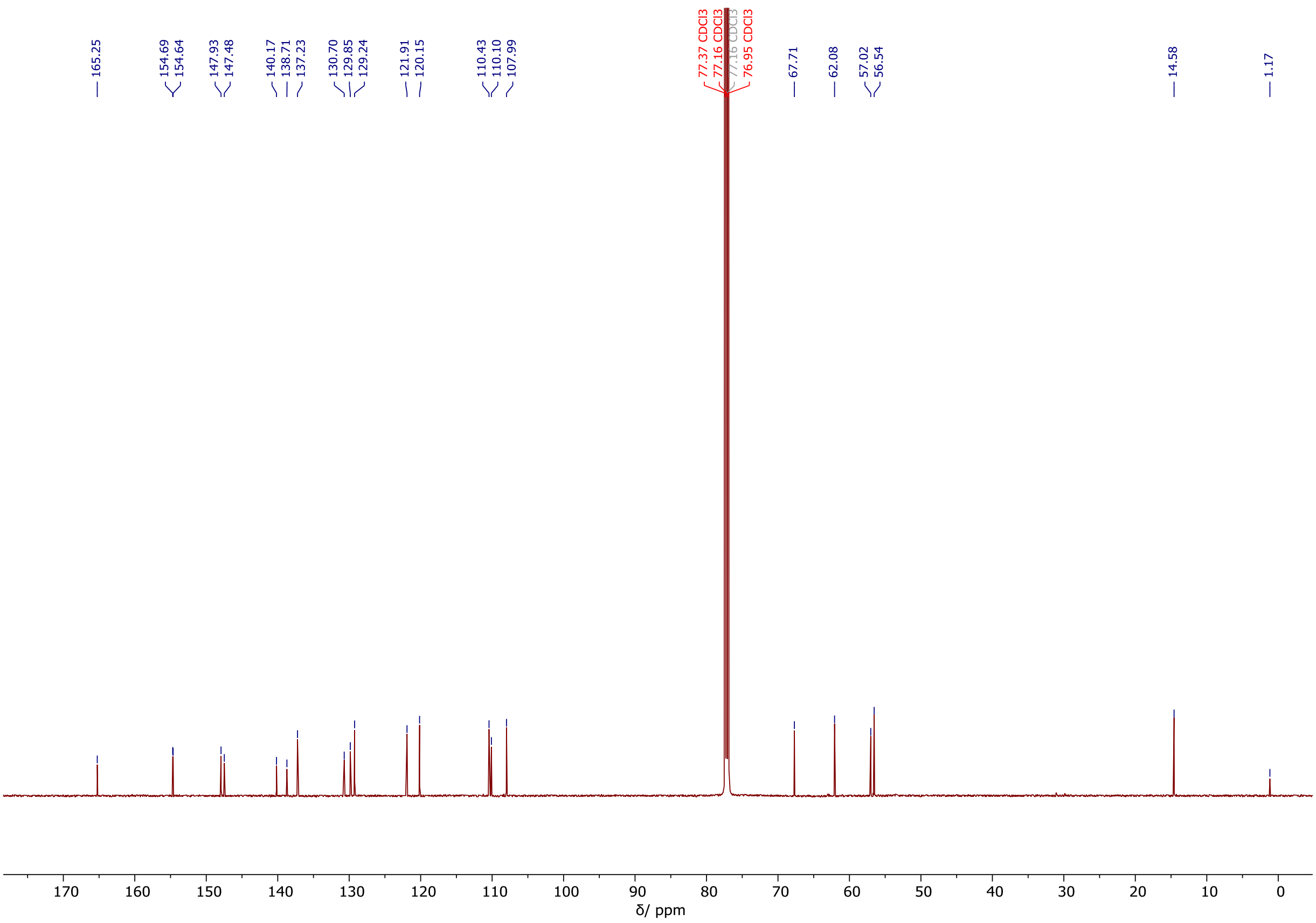

^{13}C-NMR (151 MHz, $CDCl_3$, 298 K) of compound **4**

4. ^{1}H-NMR Photo-decaging Experiment

^{1}H-NMR photo-deprotection of compound **4** was conducted in the solution phase (1 mM sample in MeCN-d_3) using Thorlabs mounted LEDs irradiation at 365 nm (using M365L4) in a Thorlabs cuvette holder (CVH100/MM). Photo-decaging experiments were monitored at known time intervals. The LEDs were supplied with 1 A current using Thorlabs T-cub LED driver (LEDD1B). The percentage deprotection was then calculated by using the Mestrenova software to integrate peaks corresponding to analogous protons on compound **2a** and the photo-caged compound **1** using equation S1:

$$\% \, conversion = \frac{I_b}{I_a + I_b} \times 100$$

(Equation S1)

where I_a and I_b are the integrals of analogous protons on **2a** and the photo-caged compound **4** respectively.

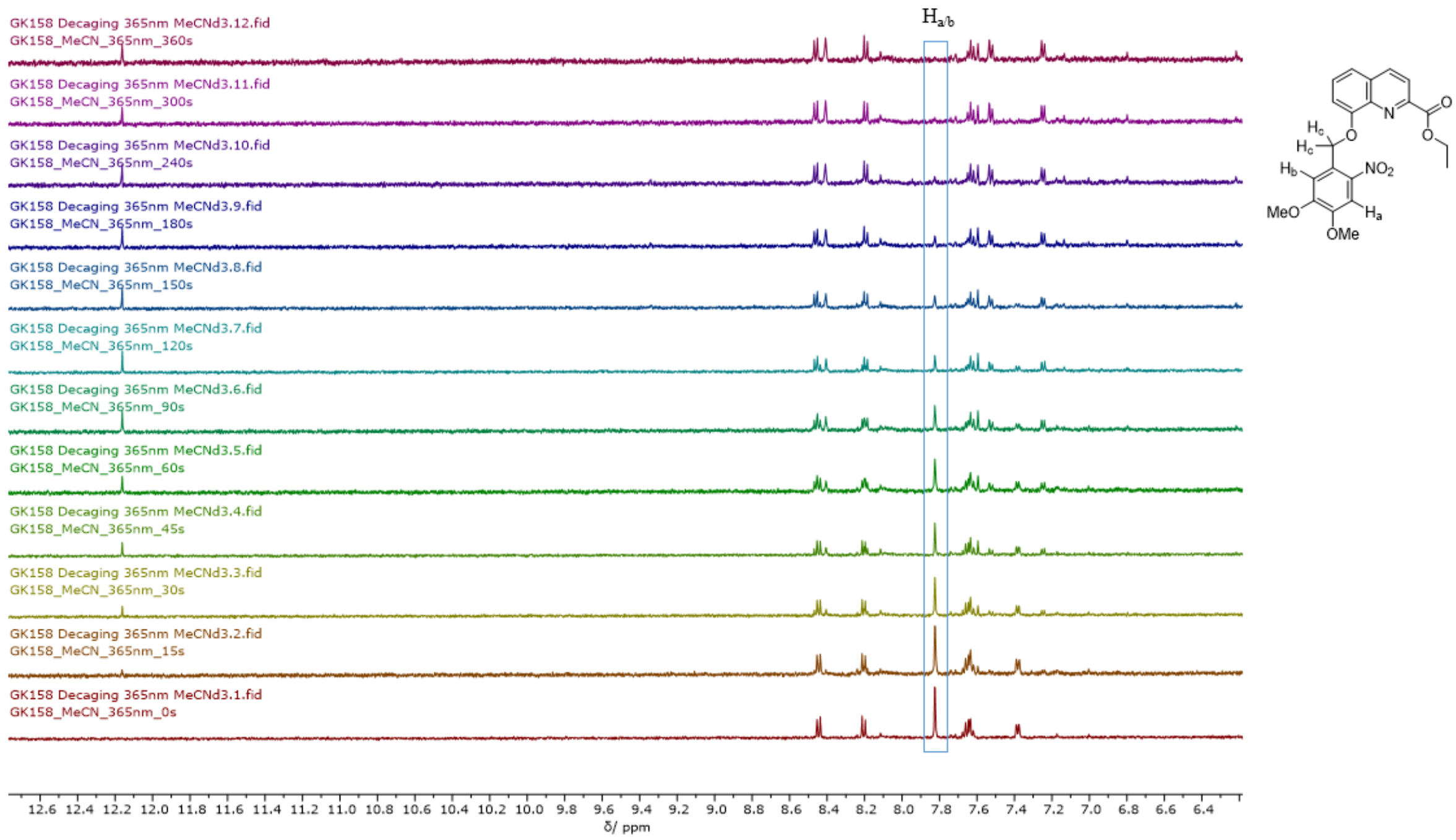

Figure S1: ^{1}H-NMR spectra for photodecaging of compound **4** using 365 nm light from 12.6- 6.4 ppm, signals in the blue box show the signals from the cage decreasing over longer irradiation times.

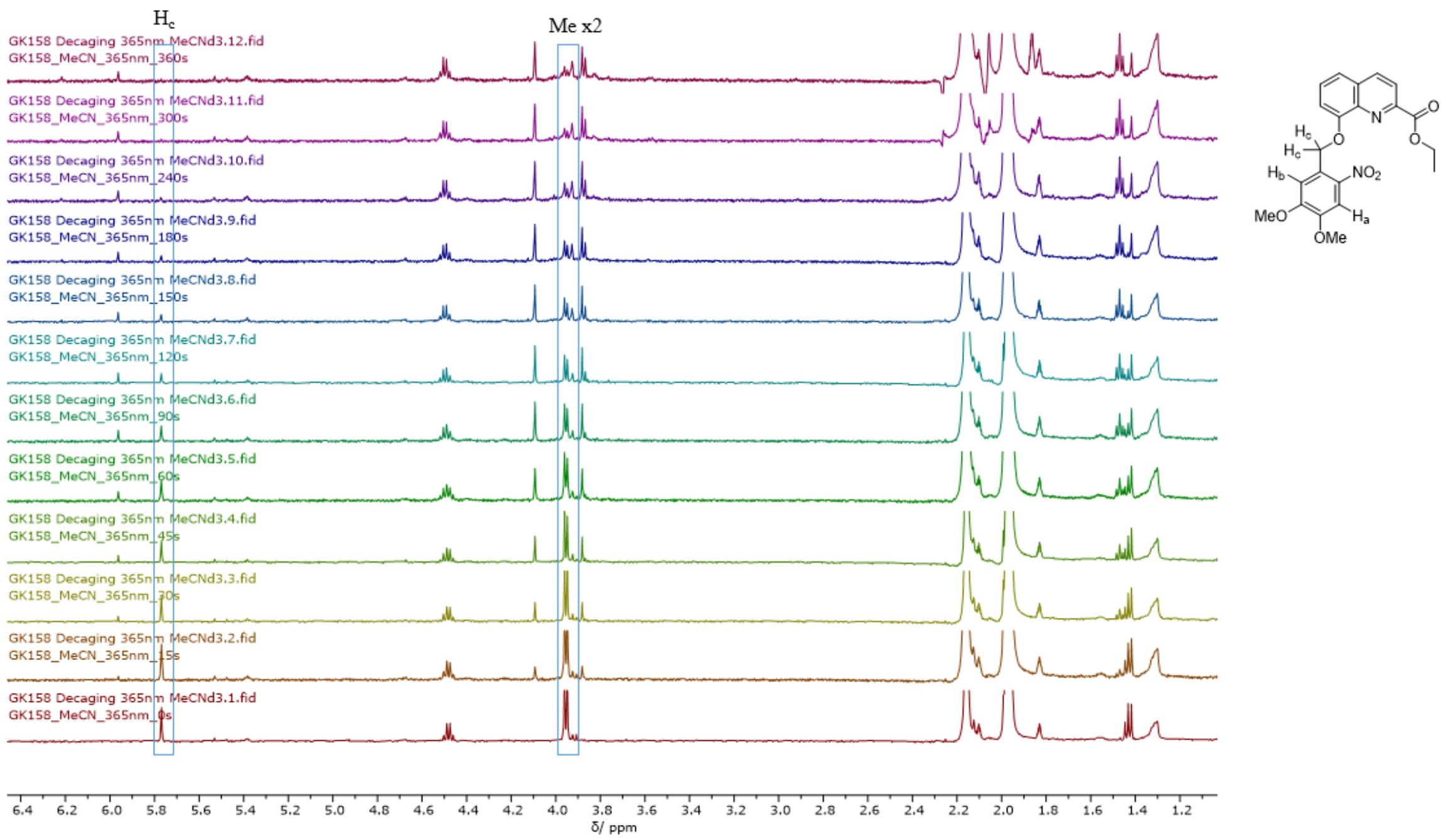

Figure S2: Truncated ^{1}H-NMR spectra for photodecaging of compound **4** using 365 nm light from 6.4-1.2 ppm, signals in the blue boxes show the signals from the cage decreasing over longer irradiation times.

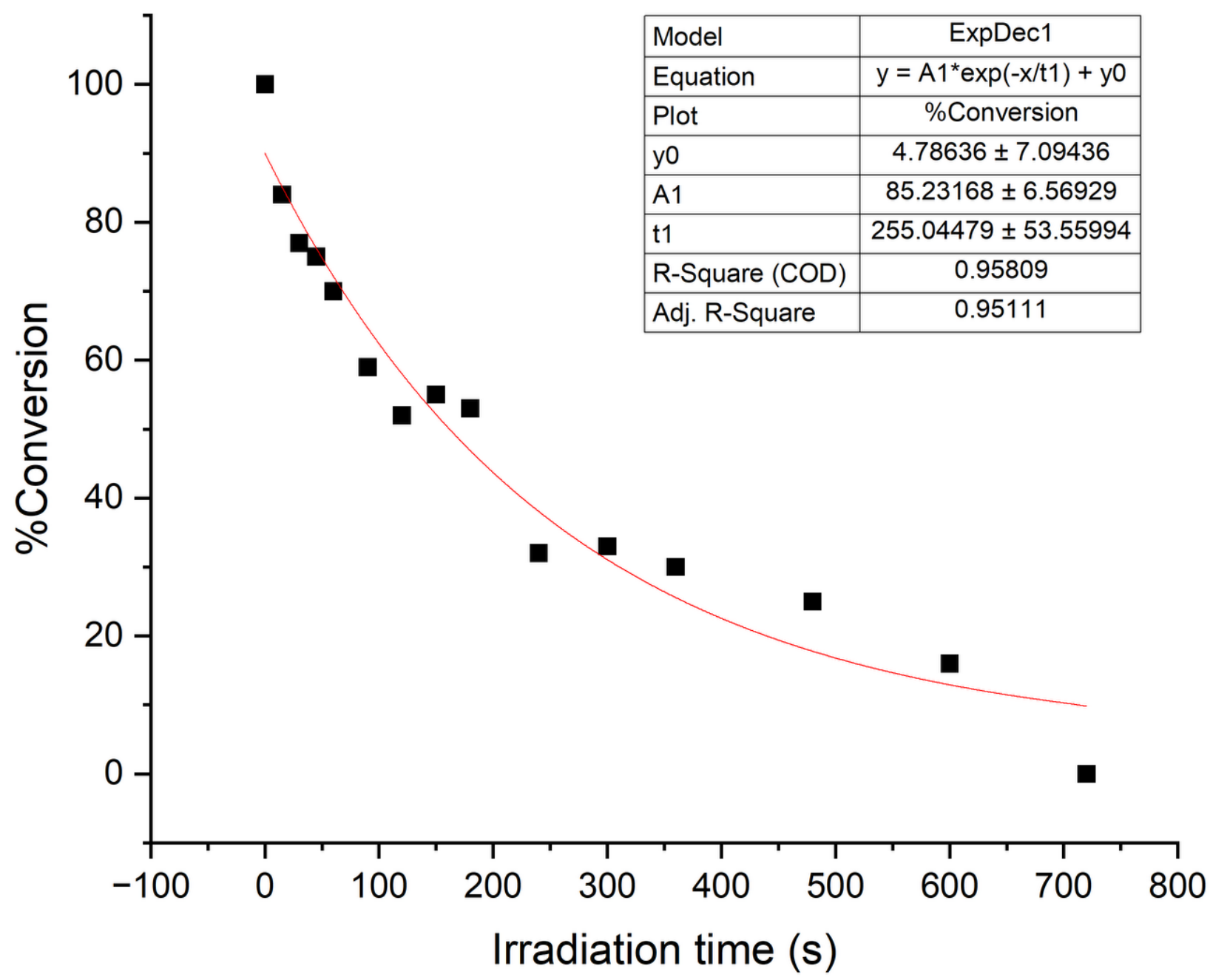

Figure S3: Plot of %conversion against the irradiation time for the ^{1}H-NMR photodecaging of compound **4** with 365 nm light, following the proton signals at 5.96 ppm and 5.77 ppm.

5. Mass Spectrometry Data

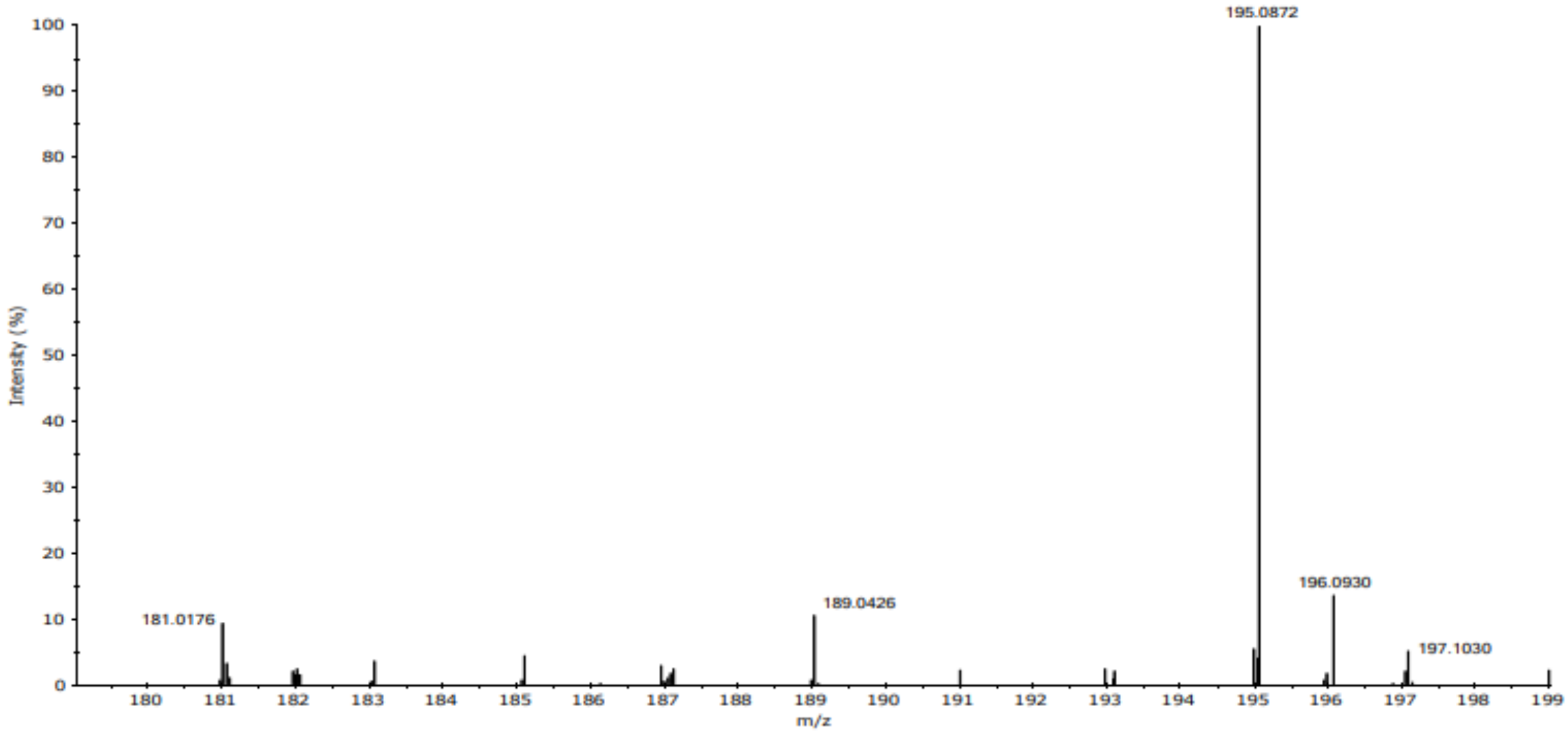

Theoretical Spectrum for C10H7NO3, Minimum Abundance 0.01%

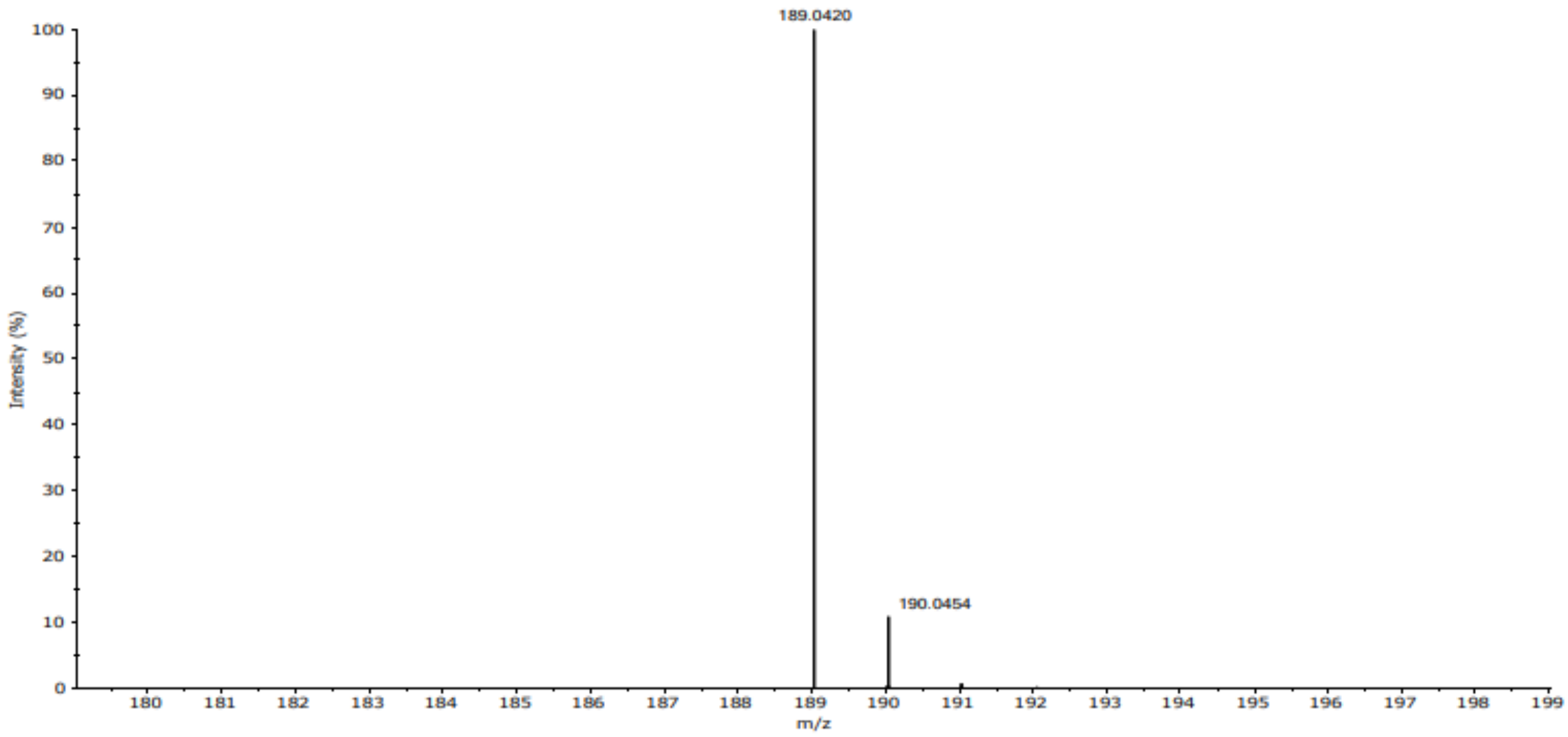

Figure S4: HRMS spectrum for compound **1**, obtained from the enzyme cleavage of compound **2a**. HRMS-ESI (m/z) Calculated for $C_{10}H_6NO_3$ $[M]^+$= 189.0420, found $[M]^+$ = 189.0426.

6. UV-vis Binding Titrations

UV-vis binding titrations for the host **1**, **2** and **4a** were conducted in methanol at 5 µM. Incremental equivalents of the guest $Ca(NO_3)_2$ (0.1 mM) were titrated into the host solution at 298K. The guest solution was made up using the host solution to prevent dilution of the host concentration. For **2a** and **1**, plateau at 0.5 equivalents is indicative of formation of a 2:1 ligand-calcium complex, with stepwise association constants too high to determine (~>10^7 M^{-1}). In contrast, 4,5-dimethoxy-2-nitrobenzyl caged **4** exhibited no binding under otherwise identical titration experimental conditions.

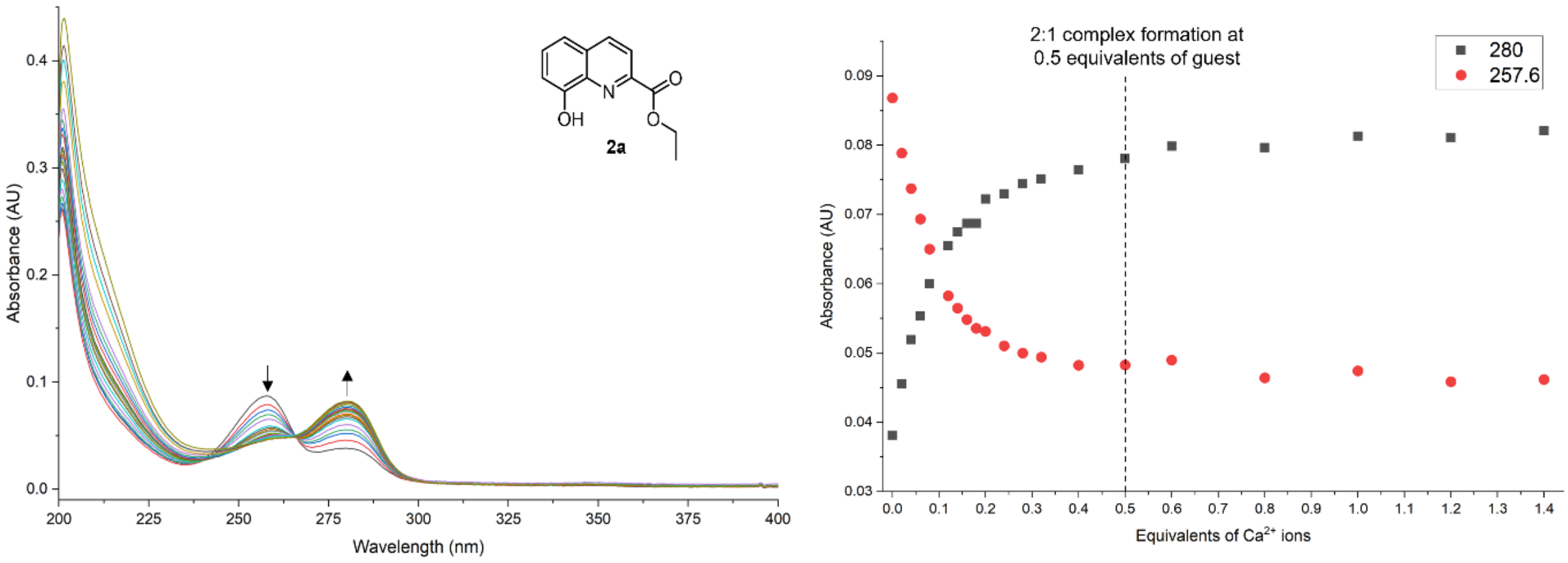

Figure S5: UV-vis spectroscopic titration performed on compound **2a** (5 µM) upon addition of increasing amounts of $Ca(NO_3)_2$ (0-1.4 equivalents).

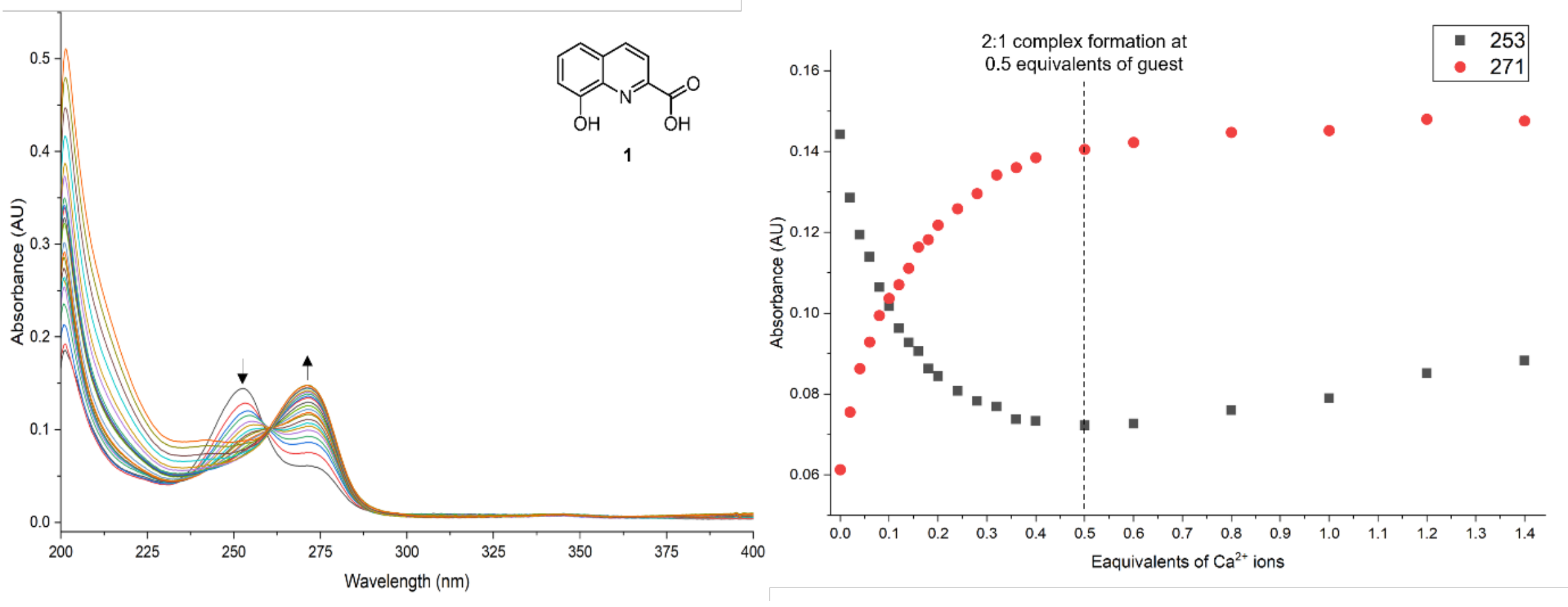

Figure S6: UV-vis spectroscopic titration performed on compound **1** (5 µM) upon addition of increasing amounts of $Ca(NO_3)_2$ (0-1.4 equivalents).

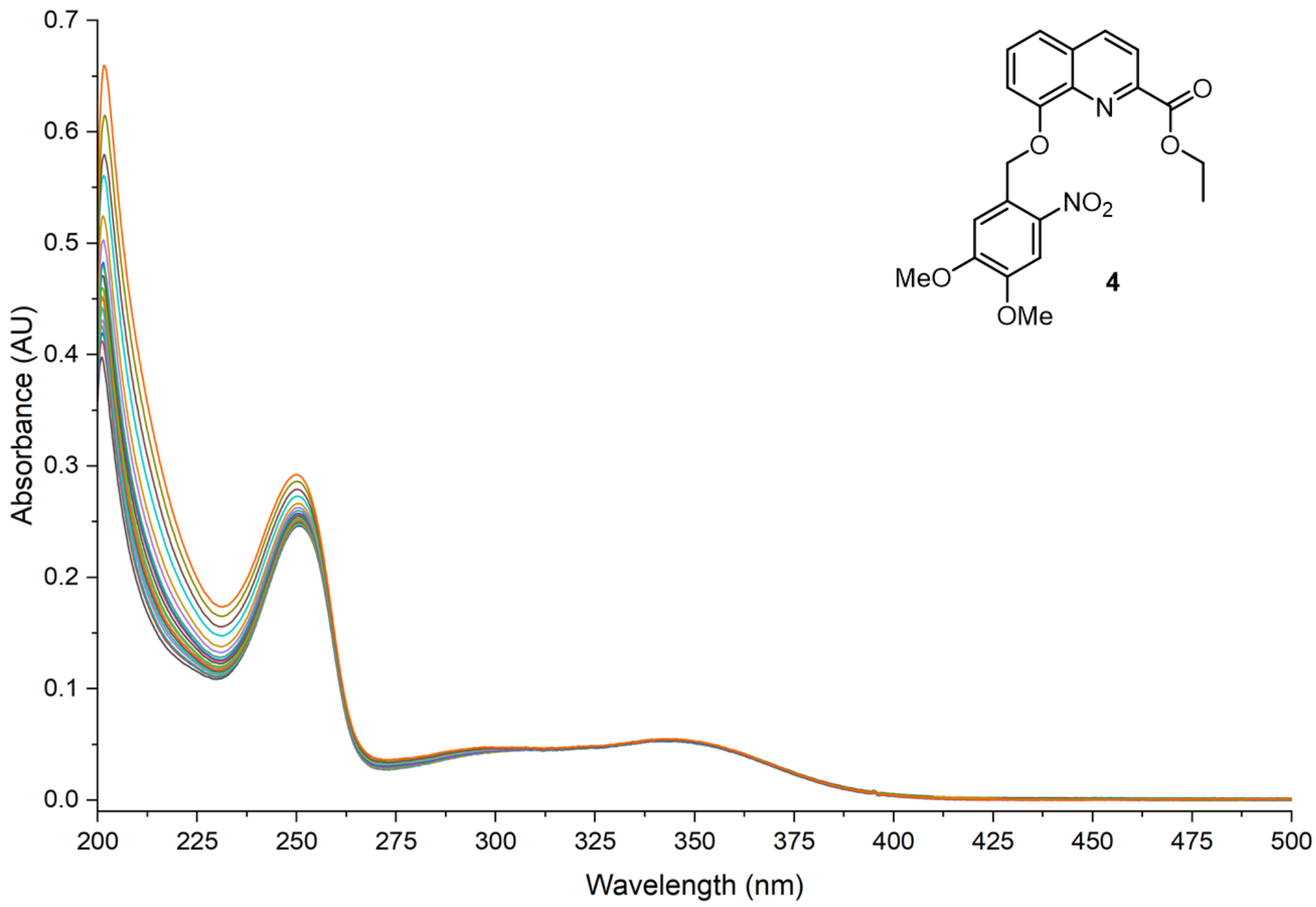

Figure S7: UV-vis spectroscopic titration performed on compound **4** (5 µM) upon addition of increasing amounts of $Ca(NO_3)_2$ (0-1.4 equivalents).

7. Fura-2 Assay Ca^{2+} Transport Experiments

Vesicle preparation in Fura-2 assay:

A thin film of lipid (1-palmitoyl-2-oleoyl-*sn*-3-phosphatidylcholine) was formed by evaporating a chloroform solution on a rotary evaporator (20 °C) and then under high vacuum for 6 hours. The lipid film was hydrated by vortexing with the prepared buffer (100 mM NaCl, 10 mM HEPES, 314 µM Fura-2 pentapotassium salt and 10 µM EDTA at pH 7.0). The lipid suspension was then subjected to 5 freeze-thaw cycles using liquid nitrogen and a water bath (40 °C), followed by extrusion 19 times through a polycarbonate membrane (pore size 200 nm) at room temperature. Extravesicular components were removed by size exclusion chromatography on a Sephadex G-25 column with prepared buffer (100 mM NaCl, 10 mM HEPES pH 7.0). Final conditions of the LUVs (2.5 mM lipid): inside 100 mM NaCl, 10 mM HEPES, 10 µM EDTA, 314 µM Fura-2; outside 100 mM NaCl, 10 mM HEPES, pH 7.0.

Standard transport assay with Fura-2 vesicles:

In a typical experiment, the LUVs containing Fura-2 (64 µL, final concentration 80 µM lipid) were added to buffer (1906 µL of 100 mM NaCl, 10 mM HEPES, pH 7.0) at 25 °C under gentle stirring. A pulse of $CaCl_2$ (20 µL in deionised water, 100 µM) was added to generate a transmembrane calcium gradient, before the ionophore (5 µL in DMSO, 2.5 µM, unless stated otherwise) was added to initiate the experiment (t = 0), followed by calcimycin (5 mL in DMSO, 0.1 µM) at 200 s to reach maximum response. Where an enzyme (10 µL in deionised water, 3 µM) was used for cleavage, the pulse was introduced to the transporter in external buffer either prior to starting the assay (ex-situ), or it was pulsed immediately alongside it during the run (in-situ). The fluorescence emission was monitored over time at λ_{em} = 510 nm (λ_{ex} = 340/380 nm). The normalised fractional fluorescence intensity (I_{rel}) was calculated from equation S2, where R_t is the fluorescence ratio at time t, R_0 is the fluorescence ratio at time 0 s (immediately after ionophore addition) and R_C is the fluorescence ratio after the addition of calcimycin:

$$I_{rel} = \frac{R_t - R_0}{R_C - R_0}$$

(Equation S2)

The data was fitted to a pseudo-first order equation (equation S3) using OriginPro 2024, where *y* is the fractional fluorescence intensity (I_{rel}) monitored in the transport assay and *x* is time (s):

$$y = \frac{V_{max}x}{K_m + x}$$

(Equation S3)

For Hills coefficient analysis, the fluorescence intensity at the end of the transport assay, *y*, was plotted as a function of the transporter concentration (*x*/ µM). The Hill coefficient (n) and the EC_{50} value calculated by fitting to the Hill equation (equation S4):

$$y = y_0 + (y_{max} - y_0)\frac{x^n}{EC_{50} + x^n}$$

(Equation S4)

where y_0 is the fractional activity in the absence of transporter, y_{max} is the fractional activity with excess transporter and *x* is the transporter concentration in the cuvette. The resulting Hill plot is shown in Figure S8b.

Initial rates were calculated by first fitting the data to equation S4 using OriginPro 2024, where *y* is the fractional fluorescence intensity (I_{rel}) and *x* is time (s). Subsequently, equation S6 was used to obtain the initial rates (s^{-1}) and these were plotted against [**2a**]^2 (μM^2):

$$y = A_1 e^{(\frac{-x}{t_1})} + y_0$$

(Equation S5)

$$Initial\ rate\ (s^{-1}) = \frac{-A_1}{T_1}$$

(Equation S6)

The resulting initial rates plot is shown in Figure S4c.

Experiments carried out in the presence of protonophore FCCP or K^+ transporter Valinomycin were carried out using the above procedure, with the addition of FCCP (5 µL in DMSO, 100 µM), Valinomycin (5 µL in DMSO, 6.25 µM) or Gramicidin (5 µL in DMSO, 6.25 µM) added at 40s along with the $CaCl_2$ pulse.

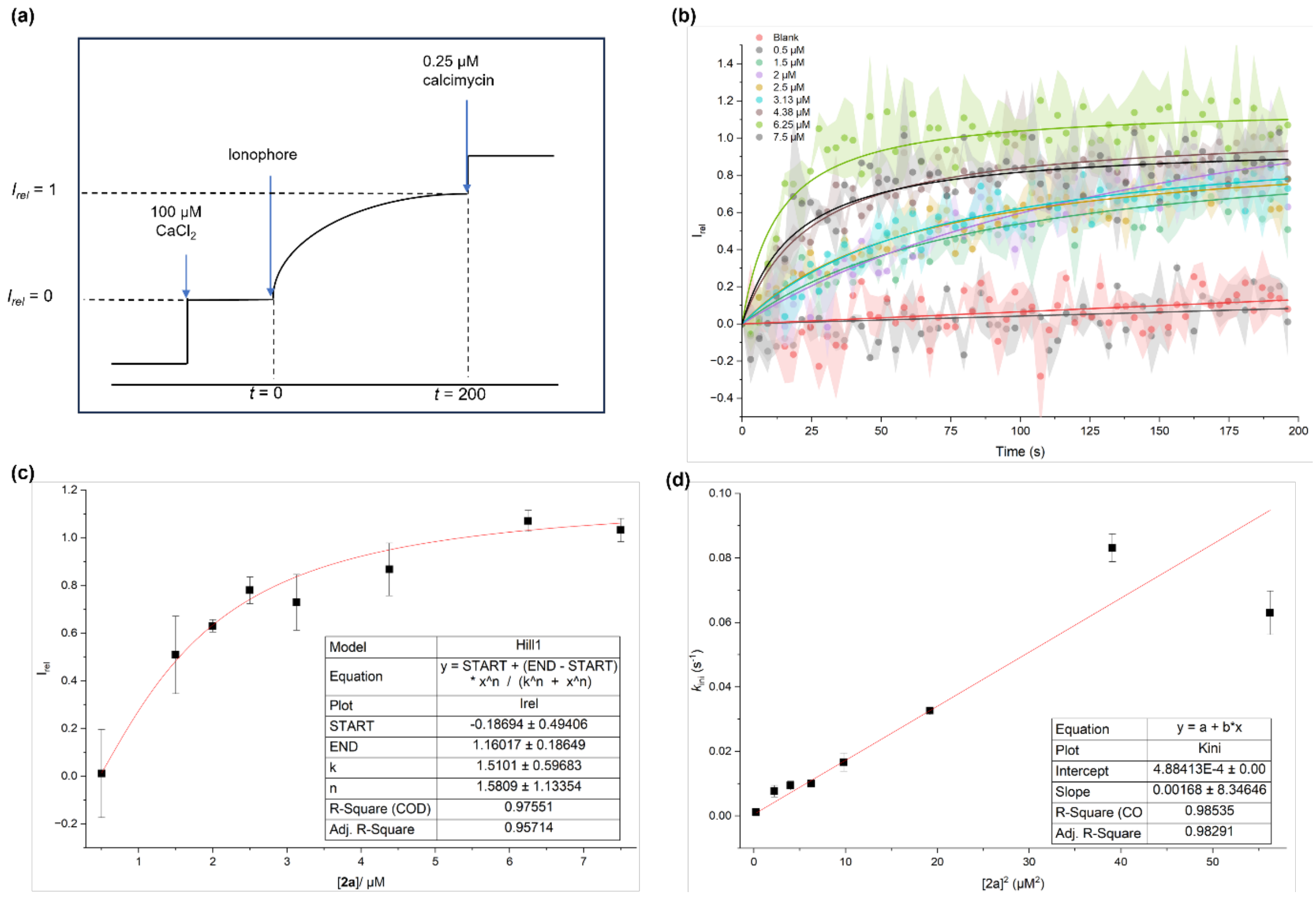

Figure S8: (a) Schematic representation of the normalisation procedure for the Fura-2 calcium transport assay. (b) Concentration of compound **2a** dependence plot showing the change in ratiometric Fura-2 emission, I_{rel} (λ_{ex} = 340/380 nm, λ_{em} = 510 nm) upon the addition of **2a** in DMSO (various concentrations) to POPC LUVs solution (80 µM) containing 314 µM Fura-2, 100 mM internal and external NaCl, 10 µM internal EDTA and 100 µM external Ca^{2+}, buffered with 10 mM HEPES to pH 7.0; (c) Dependence on fractional activities (y, the relative intensity at t= 200 s immediately prior to Ca^{2+} gradient dissipation by excess calcimycin) on concentration of **2a** (black squares), and fit to the Hill equation (red line); (d) Dependence of k_{ini} on $[\mathbf{2a}]^2$ and linear fit.

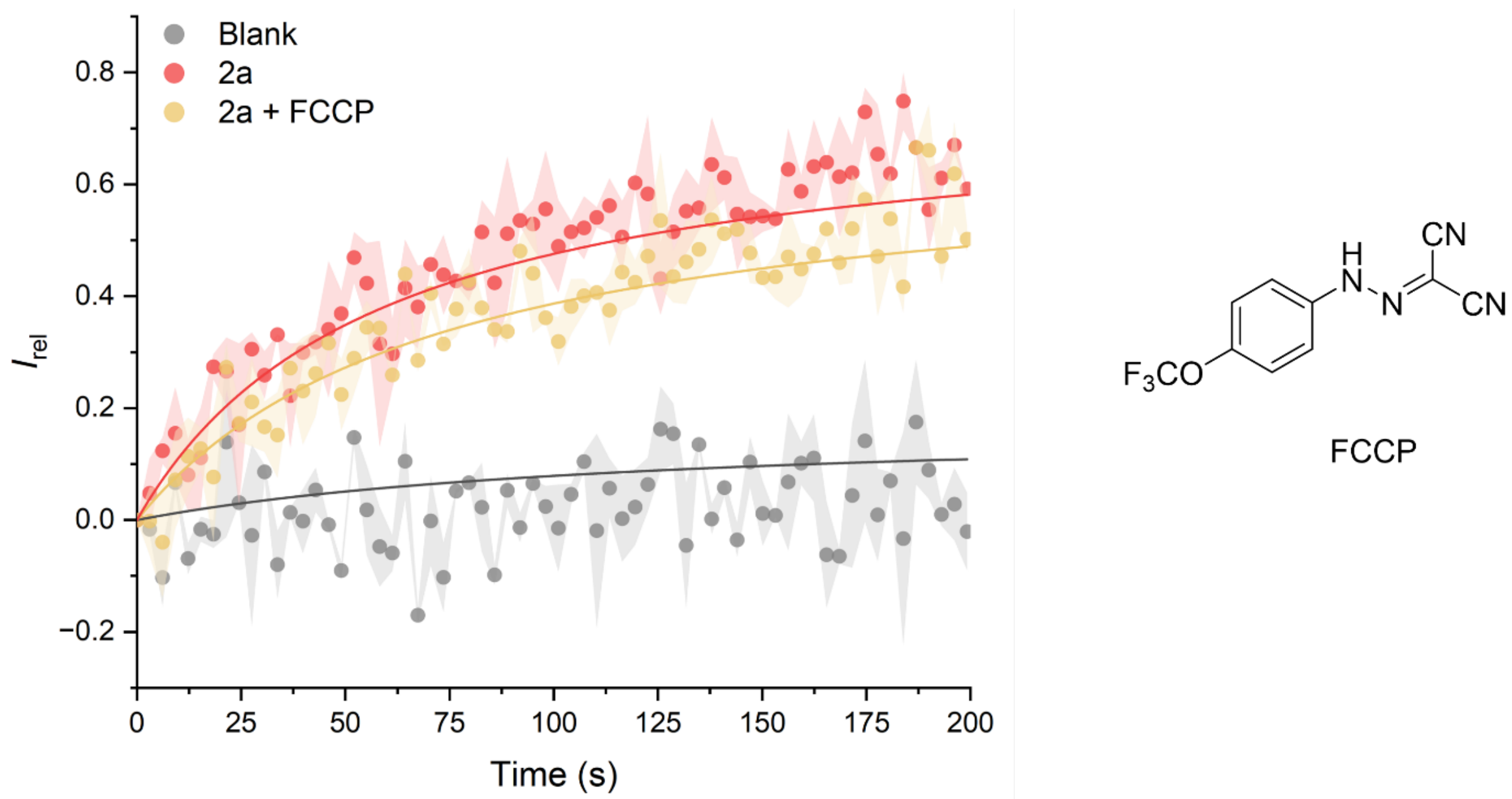

Figure S9: Change in ratiometric Fura-2 emission, I_{rel} (λ_{ex} = 340/380 nm, λ_{em} = 510 nm) upon the addition of **2a** (2.5 µM, added in 5 µL DMSO) and FCCP (0.25 µM in DMSO) in POPC LUVs (80 µM) containing 314 µM Fura-2, 100 mM NaCl, 10 µM EDTA, 10 mM HEPES, pH 7.0, suspended in 100 mM NaCl, 10 mM HEPES, pH 7.0 and 100 µM $CaCl_2$.

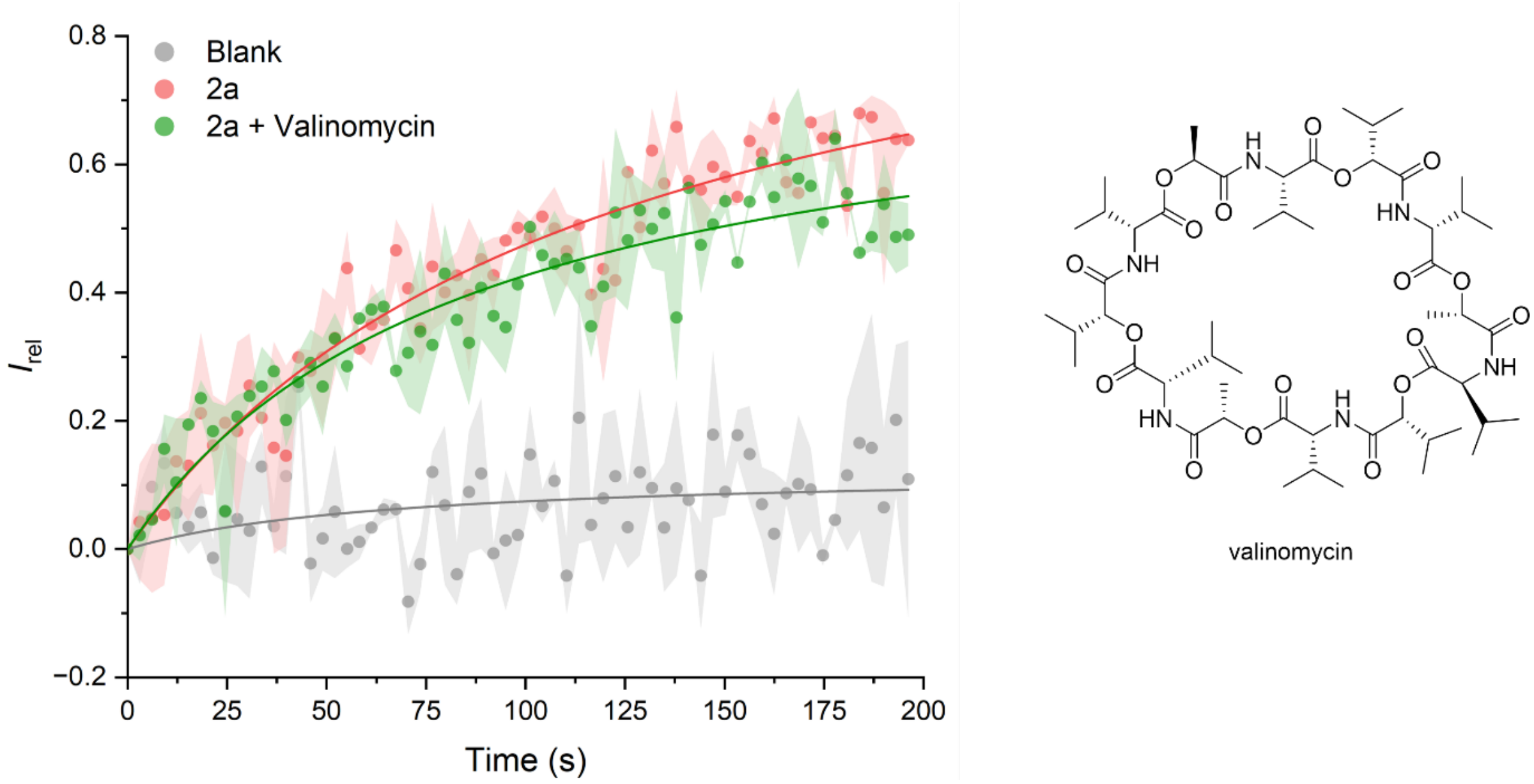

Figure S10: Change in ratiometric Fura-2 emission, I_{rel} (λ_{ex} = 340/380 nm, λ_{em} = 510 nm) upon the addition of **2a** (2.5 µM, added in 5 µL DMSO) with valinomycin (15.6 nM, added in 5 µL DMSO) in POPC LUVs (80 µM) containing 314 µM Fura-2, 100 mM KCl, 10 µM EDTA, 10 mM HEPES, pH 7.0, suspended in 100 mM KCl, 10 mM HEPES, pH 7.0 and 100 µM $CaCl_2$.

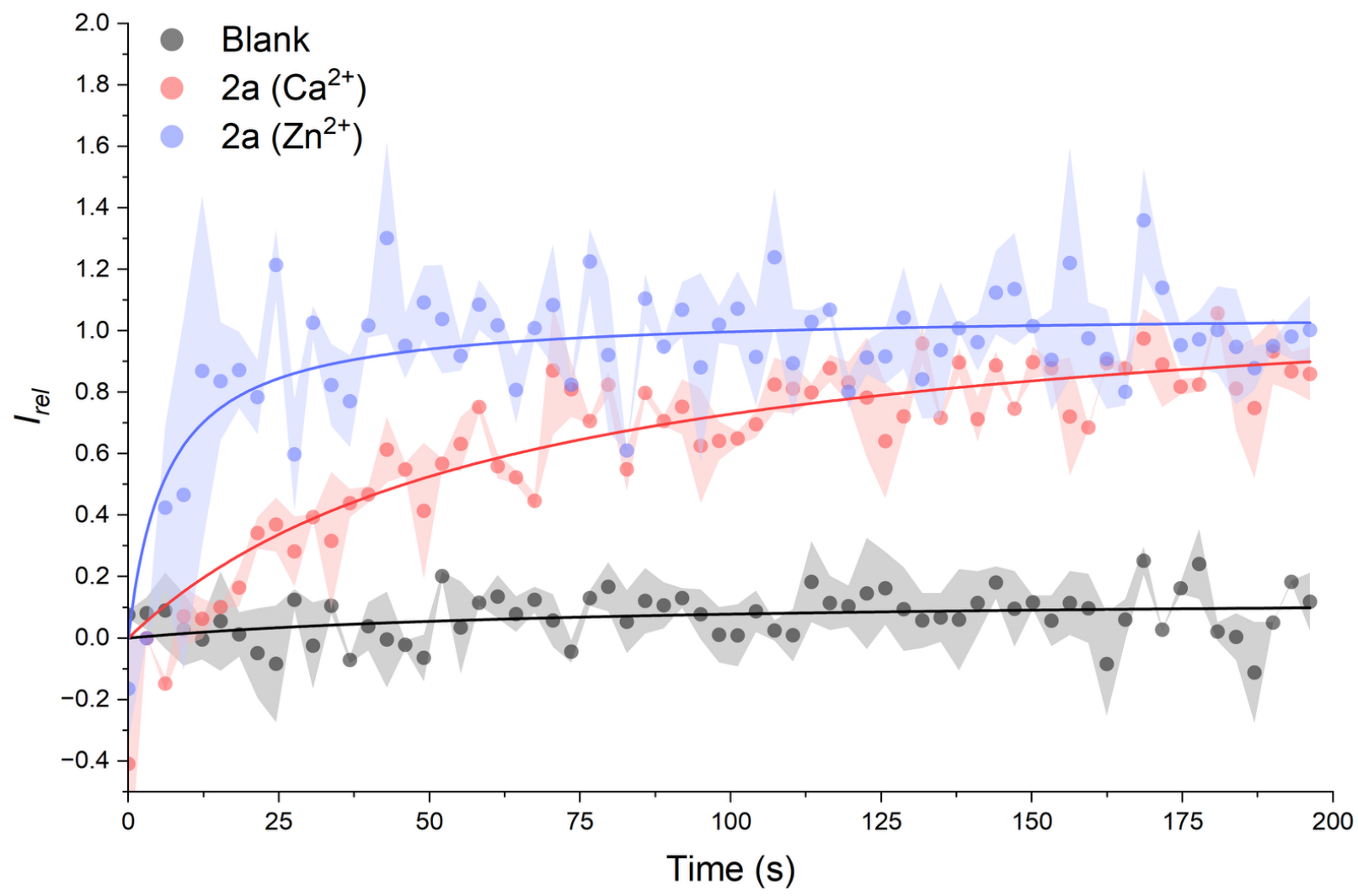

Figure S11: Change in ratiometric Fura-2 emission, I_{rel} (λ_{ex} = 340/380 nm, λ_{em} = 510 nm) upon the addition of **2a** (2.5 µM, added in 5 µL DMSO) in POPC LUVs (80 µM) containing 314 µM Fura-2, 100 mM NaCl, 10 µM EDTA, 10 mM HEPES, pH 7.0, suspended in 100 mM NaCl, 10 mM HEPES, pH 7.0 and 100 µM MCl_2 (where M= Ca^{2+}, Zn^{2+}).

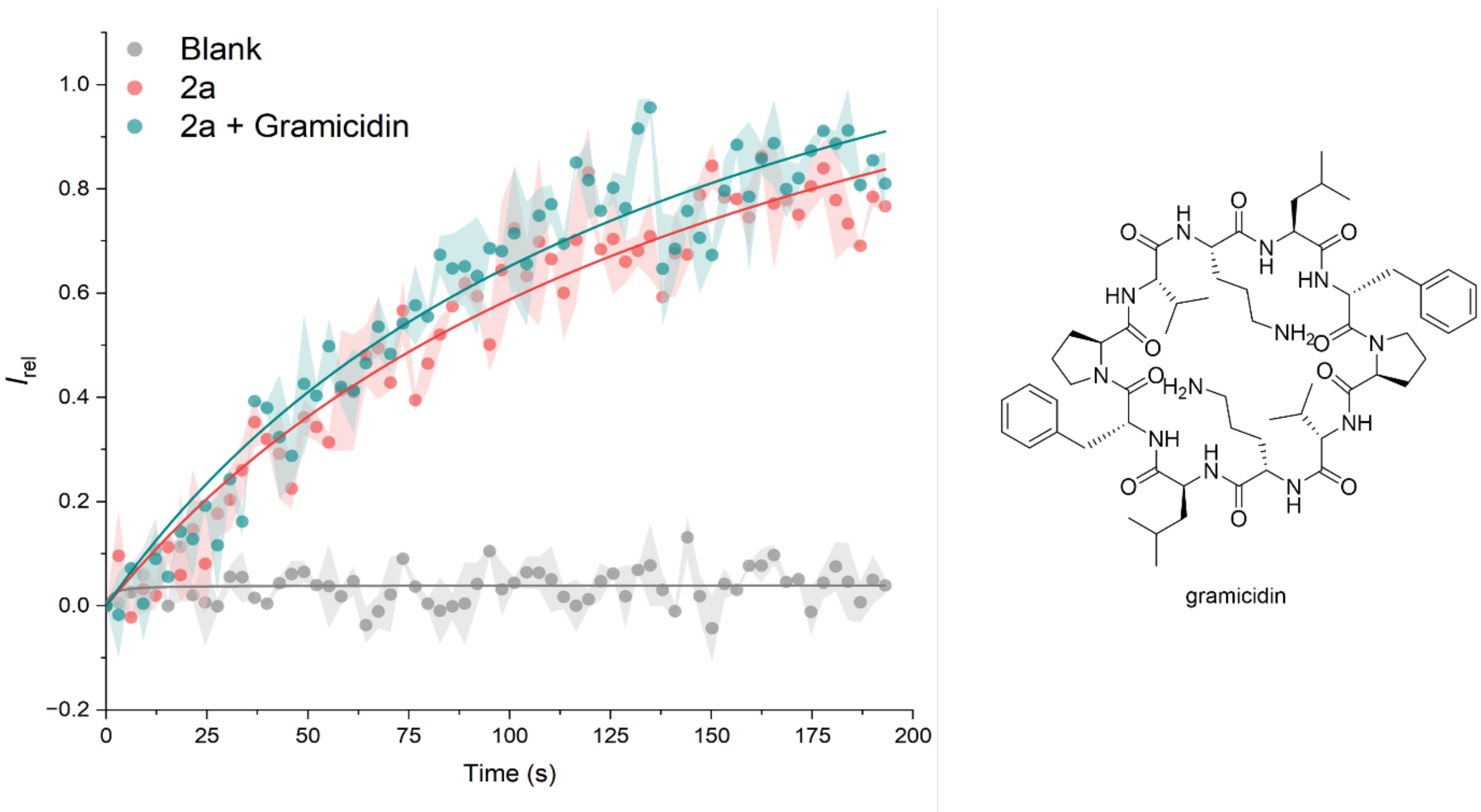

Figure S12: Change in ratiometric Fura-2 emission, I_{rel} (λ_{ex} = 340/380 nm, λ_{em} = 510 nm) upon the addition of **2a** (2.5 µM, added in 5 µL DMSO) with gramicidin (15.6 nM, added in 5 µL DMSO) in POPC LUVs (80 µM) containing 314 µM Fura-2, 100 mM NaGluconate, 10 µM EDTA, 10 mM HEPES, pH 7.0, suspended in 100 mM NaGluconate, 10 mM HEPES, pH 7.0 and 100 µM $CaCl_2$.

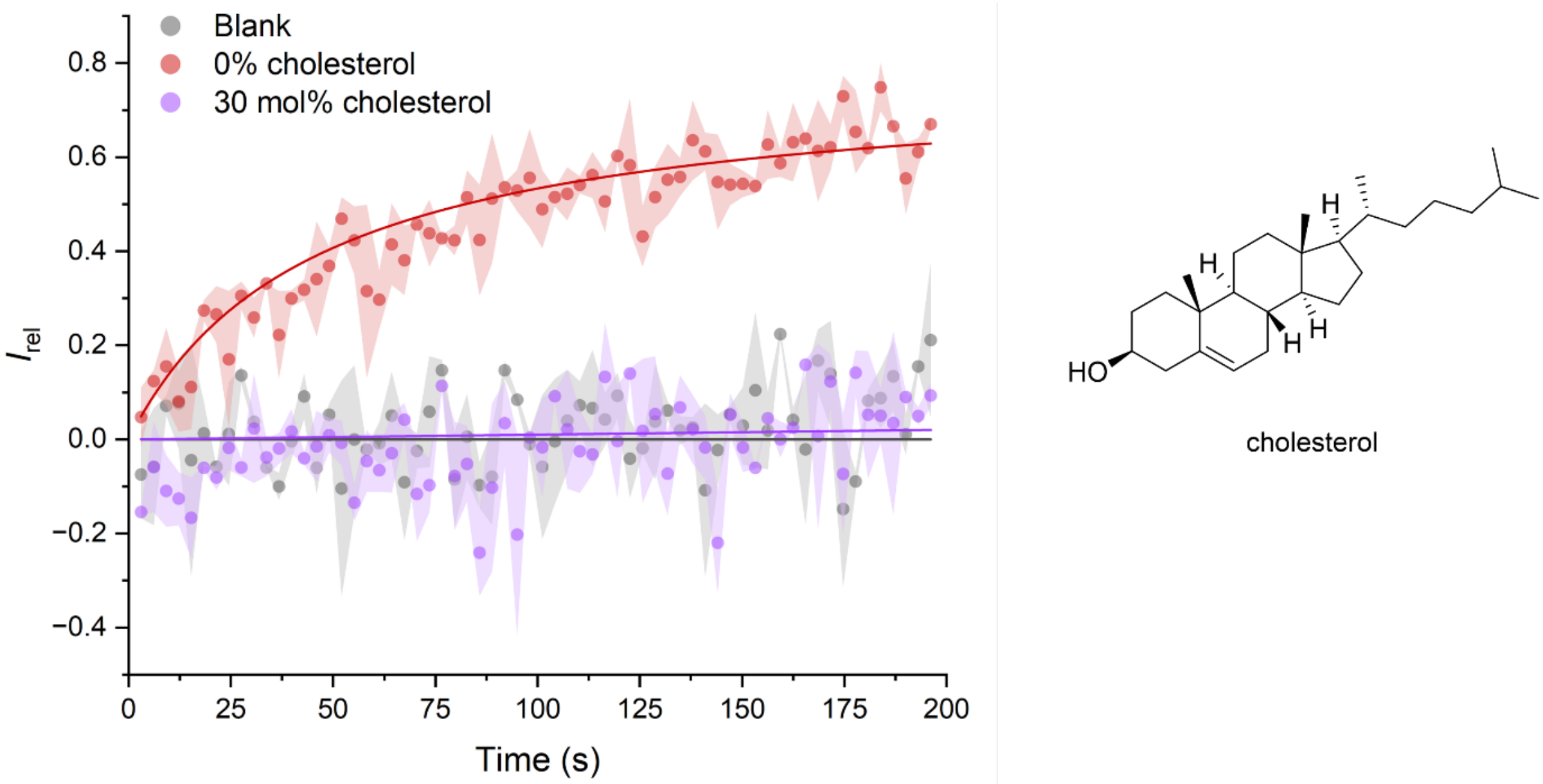

Figure S13: Change in ratiometric Fura-2 emission, I_{rel} (λ_{ex} = 340/380 nm, λ_{em} = 510 nm) upon the addition of **2a** (2.5 µM, added in 5 µL DMSO) in POPC LUVs or 7:3 POPC:cholesterol LUVs (80 µM) containing 314 µM Fura-2, 100 mM NaCl, 10 µM EDTA, 10 mM HEPES, pH 7.0, suspended in 100 mM NaCl, 10 mM HEPES, pH 7.0 and 100 µM $CaCl_2$.

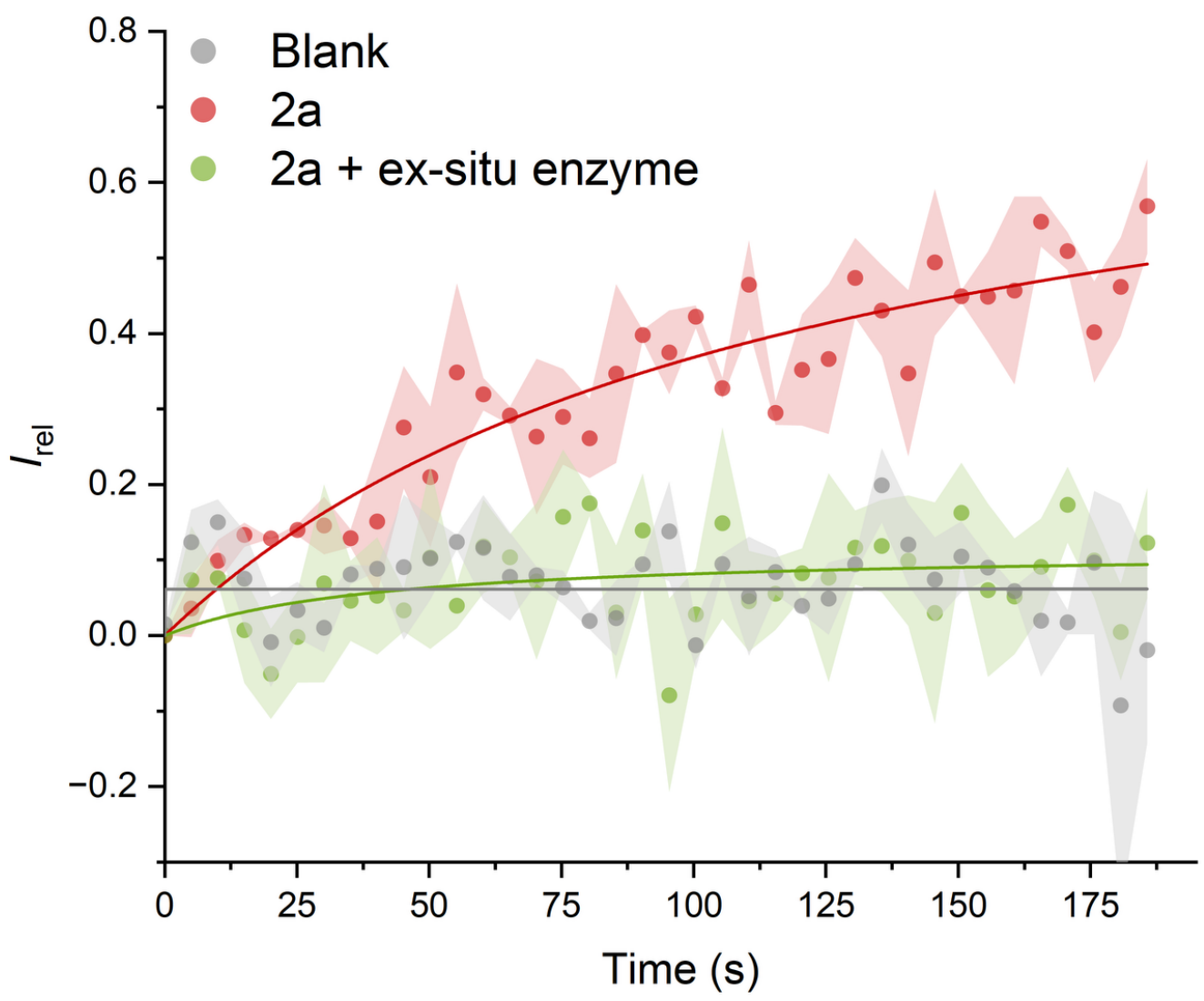

Figure S14: Change in ratiometric Fura-2 emission, I_{rel} (λ_{ex} = 340/380 nm, λ_{em} = 510 nm) upon the addition of inactivated **2a** (2.5 µM, added in 5 µL DMSO in POPC LUVs (80 µM) containing 314 µM Fura-2, 100 mM NaCl, 10 µM EDTA, 10 mM HEPES, pH 7.0, suspended in 100 mM NaCl, 10 mM HEPES, pH 7.0 and 100 µM $CaCl_2$. Inactivated **2a** was obtained by incubating **2a** with porcine liver esterase (3.0 µM, added in 5 µL water) in external buffer prior to addition of LUVs (ex-situ enzyme cleavage).

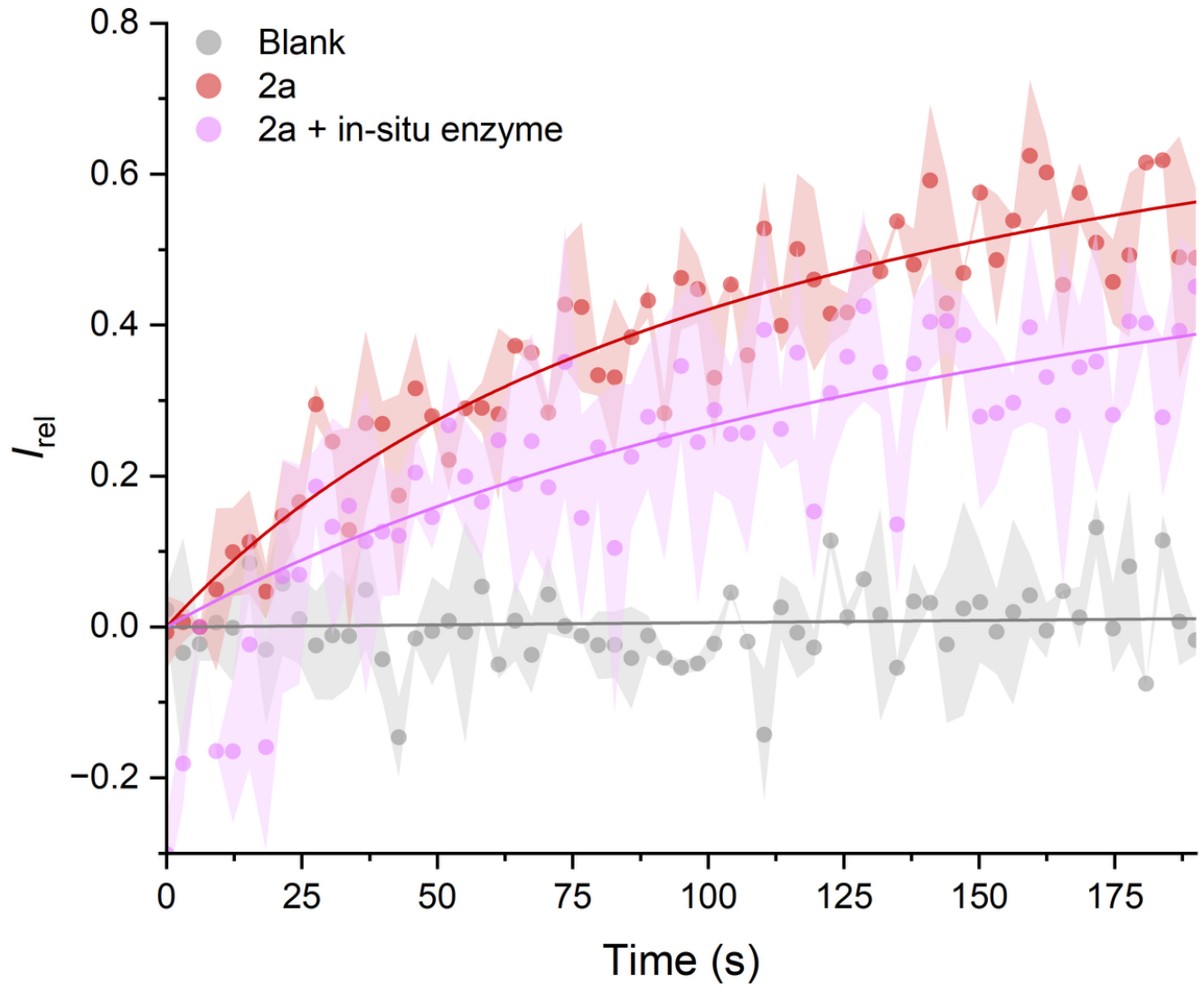

Figure S15: Change in ratiometric Fura-2 emission, I_{rel} (λ_{ex} = 340/380 nm, λ_{em} = 510 nm) upon the addition of a pulse of **2a** (2.5 µM, added in 5 µL DMSO) with and without immediate addition of porcine liver esterase (3.0 µM, added in 5 µL water) in POPC LUVs (80 µM) containing 314 µM Fura-2, 100 mM NaCl, 10 µM EDTA, 10 mM HEPES, pH 7.0; suspended in 100 mM NaCl, 10 mM HEPES, pH 7.0 and 100 µM $CaCl_2$.

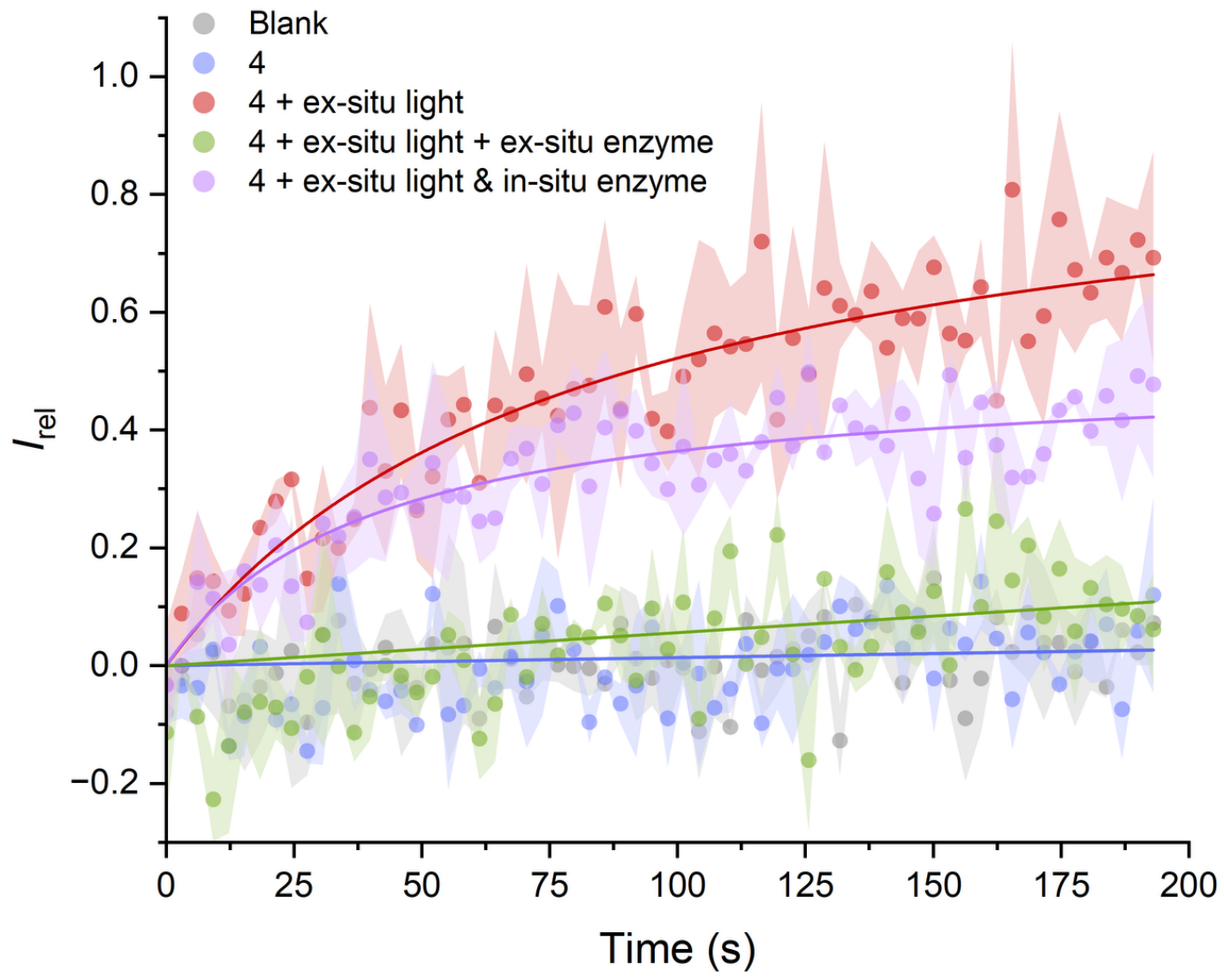

Figure S16: Change in ratiometric Fura-2 emission, I_{rel} (λ_{ex} = 340/380 nm, λ_{em} = 510 nm) upon the addition of a photo-irradiated (365 nm, 25 s) sample **4** (2.5 µM, added in 5 µL DMSO) in external buffer (ex-situ irradiation), followed by the ex-situ (prior to the start of the assay, in the absence of LUVs) and in-situ (during the assay run, in the presence of LUVs) addition of porcine liver esterase (3.0 µM, added in 5 µL water) in POPC LUVs (80 µM) containing 314 µM Fura-2, 100 mM NaCl, 10 µM EDTA, 10 mM HEPES, pH 7.0; suspended in 100 mM NaCl, 10 mM HEPES, pH 7.0 and 100 µM $CaCl_2$.

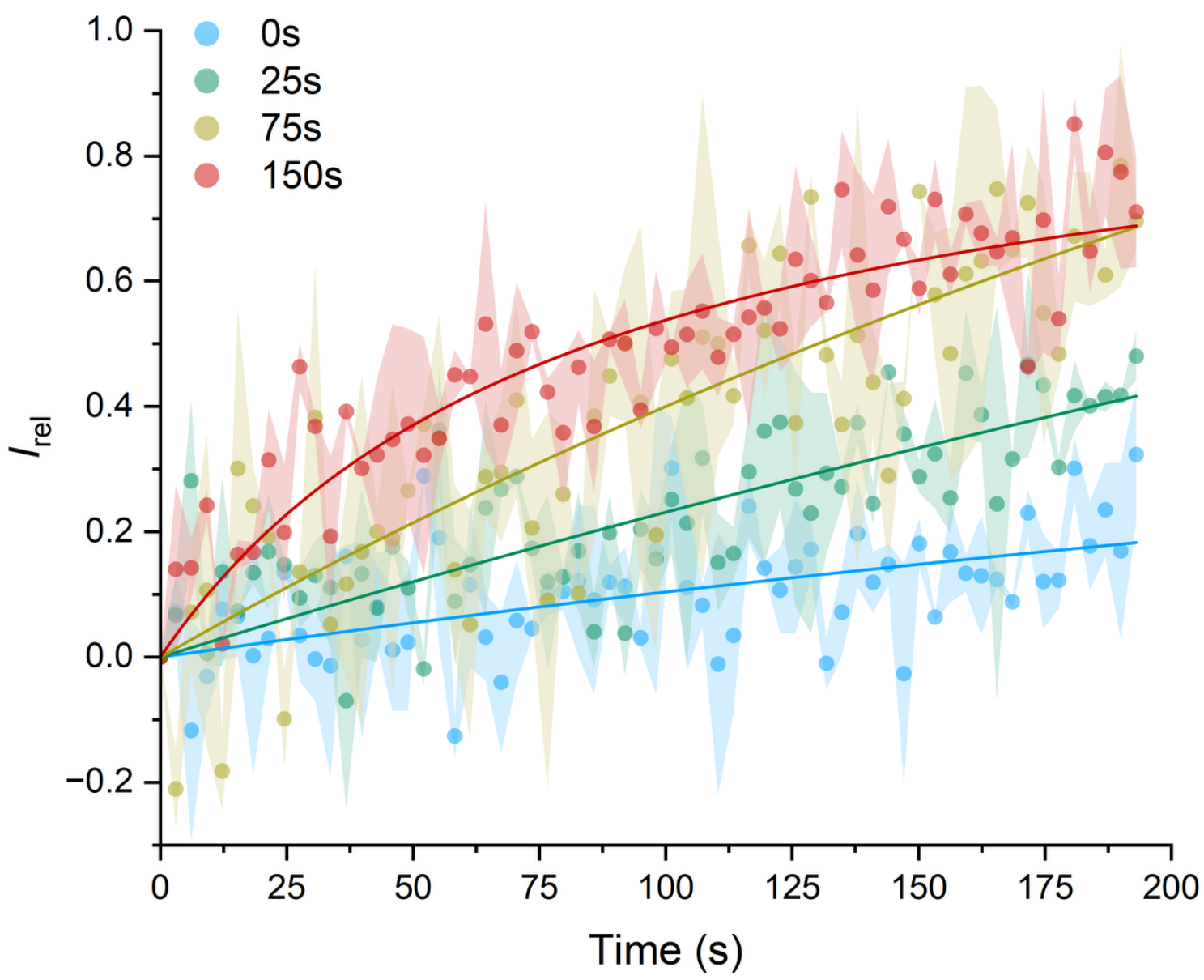

Figure S17: Change in ratiometric Fura-2 emission, I_{rel} (λ_{ex} = 340/380 nm, λ_{em} = 510 nm) upon the incubation of photo-irradiated (for varied time lengths) sender POPC LUVs (80 µM), containing compound **4** (2.5 µM, added in 5 µL DMSO), and POPC LUVs (80 µM) containing 314 µM Fura-2, 100 mM NaCl, 10 µM EDTA, 10 mM HEPES, pH 7.0; suspended in 100 mM NaCl, 10 mM HEPES, pH 7.0 and 100 µM $CaCl_2$.

8. HPTS Assay Studies

Vesicle preparation in HPTS assay:

A thin film of lipid (1-palmitoyl-2-oleoyl-*sn*-3-phosphatidylcholine) was formed by evaporating a chloroform solution on a rotary evaporator (20 °C, and then under high vacuum for 6 hours. The lipid film was hydrated by vortexing with the prepared buffer (100 mM $CaCl_2$, 10 mM HEPES, 1 mM 8-hydroxypyrene-1,3,6-trisulfonic acid trisodium salt (HPTS), pH 7.0). The lipid suspension was then subjected to 5 freeze-thaw cycles using liquid nitrogen and a water bath (40°C), followed by extrusion 19 times through a polycarbonate membrane (pore size 200 nm) at rt. Extra-vesicular components were removed by size exclusion chromatography on a Sephadex G-25 column with 100 mM NaCl, 10 mM HEPES, pH 7.0. Final conditions: LUVs (2.5 mM lipid); inside 100 mM $CaCl_2$, 10 mM HEPES, 1 mM HPTS, pH 7.0; outside: 100 mM $CaCl_2$, 10 mM HEPES, pH 7.0.

For HPTS assay experiments where different buffer conditions were used, the preparation procedure used was unchanged.

Transport assay with HPTS vesicles:

In a typical experiment, the LUVs containing HPTS (25 µL, final lipid concentration 31.3 mM) were added to buffer (1950 µL of 100 mM $CaCl_2$, 10 mM HEPES, pH 7.0) at 25°C under gentle stirring. A pulse of NaOH (20 µL, 5 mM) was added at 40 s to initiate the experiment. At 90 s the test transporter (5 µl in DMSO) was added, followed by detergent (25 µL of Triton X-100 in 7:1 (v/v) H2O-DMSO) at 290 s to calibrate the assay.

The fractional fluorescence intensity (I_{rel}) was calculated from equation S7, where R_t is the fluorescence ratio at time *t*, R_0 is the fluorescence ratio at time *0*, and R_d is the fluorescence ratio after the addition of detergent.

$$I_{rel} = \frac{R_t - R_0}{R_d - R_0}$$

(Equation S7)

Experiments carried out in the presence of protonophore FCCP were carried out using the above procedure, with the addition of FCCP (100 µM, 5 µL DMSO) added at 55 s along with the base pulse. At this concentration, FCCP does not cause appreciable dissipation of the transmembrane pH gradient alone.

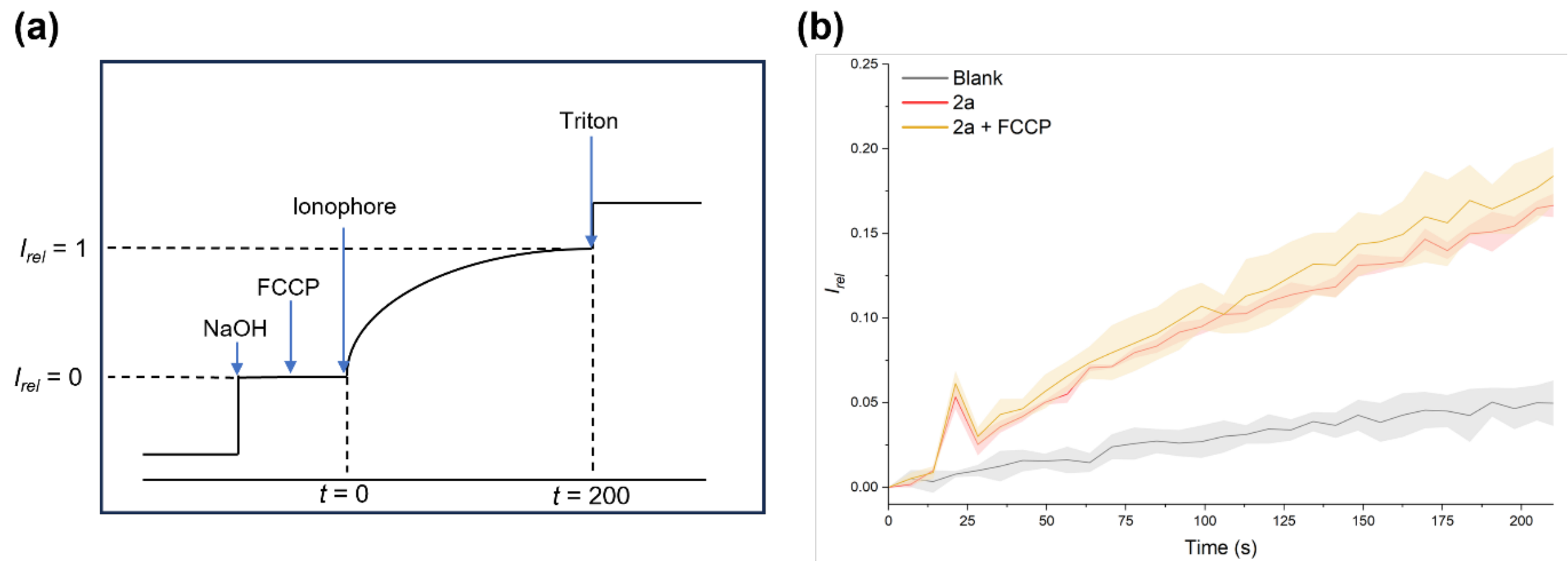

Figure S18: Change in HPTS emission, I_{rel} (λ_{ex}= 405/465 nm, λ_{em}= 510 nm) upon the addition of **2a** (5 µM in DMSO) with and without FCCP (0.25 µM in DMSO) in POPC LUVs (31.3 µM) containing 1 mM HPTS, 100 mM $CaCl_2$, 10 mM HEPES, pH 7.0; suspended in 100 mM $CaCl_2$, 10 mM HEPES, pH 7.0. A pH gradient was generated by addition of 5 mM NaOH.

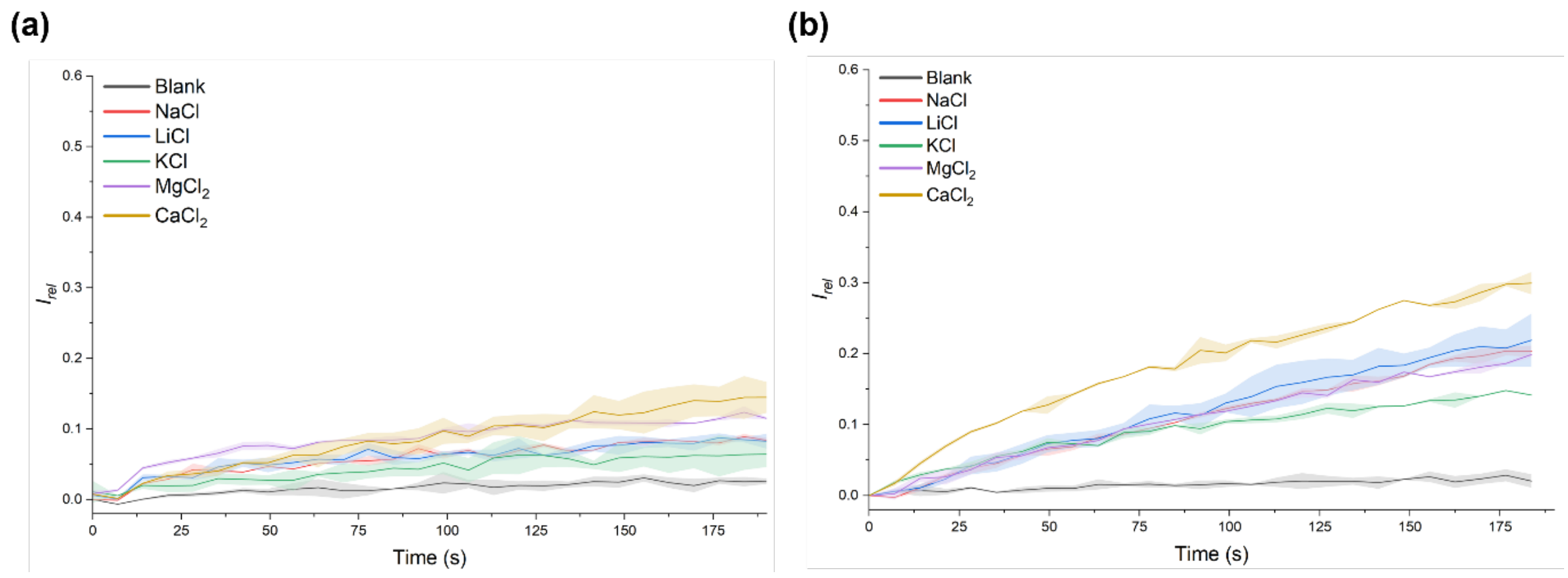

Figure S19: Change in HPTS emission, I_{rel} (λ_{ex}= 405/465 nm, λ_{em}= 510 nm) upon the addition of **2a** (2.5 µM on the left and 5 µM on the right) with FCCP (0.25 µM in DMSO) in POPC LUVs (31.3 µM) containing 1 mM HPTS, 100 mM $NaCl_2$, 10 mM HEPES, pH 7.0; suspended in 100 mM MCl/MCl_2, 10 mM HEPES, pH 7.0, where M= Li^+, Na^+, K^+, Mg^{2+}, Ca^{2+}. A pH gradient was generated by addition of 5 mM NaOH.

These experiments reveal selectivity for Ca^{2+} over Li^+, Na^+, K^+, Mg^{2+}, which are transported to a minimal extent with comparable activity in this assay.

9. Calcium Ion Selective Electrode (ISE) Assay Experiments

A thin film of lipid (1-palmitoyl-2-oleoyl-*sn*-3-phosphatidylcholine) lipids was formed by evaporation of chloroform solutions on a rotary evaporator (20 °C) and then dried under high vacuum for at least 6 hours. The lipid film was hydrated by vortexing with the prepared buffer (500 mM $CaCl_2$, buffered to pH 7.0 with 5 mM sodium phosphate salts). The lipid suspension was then subjected to 5 freeze-thaw cycles using liquid nitrogen and a water bath (40°C), followed by extrusion 19 times through a polycarbonate membrane (pore size 200 nm) at room temperature. Extra-vesicular components were removed by size exclusion chromatography on a Sephadex G-25 column eluted with buffer (500 mM NaCl, buffered with phosphate salts to pH 7.0).

The vesicles suspension was then diluted to 30 mL in 500 mM NaCl, pH 7.0 phosphate buffer. Final conditions after dilution: LUVs (1 mM lipid); inside 500 mM $CaCl_2$, buffered to pH 7.0 with 5 mM sodium phosphate salts; outside 500 mM NaCl, buffered with phosphate salts to pH 7.0. Vesicles were stored at ~4°C and used within 24 hrs. The solution was warmed to r.t. prior to use.

Electrode calibration:

The calcium selective electrode (Thermo Scientific™ Orion™ Sure-Flow® Combination Calcium Electrode ISE) was soaked in 0.01 M calcium standard solution for extended periods (>1-2 hr) until readings were stable. At the start of the experiment, the electrode was calibrated by recording the potential of known calcium concentrations. The readings (mV) were converted to calcium activity using the modified Nernst equation S8, where y is the calcium activity, *x* is the potential (mV) and a and b are parameters to be determined and c~0.

$$y = a - bln(x + c)$$

(Equation S8)

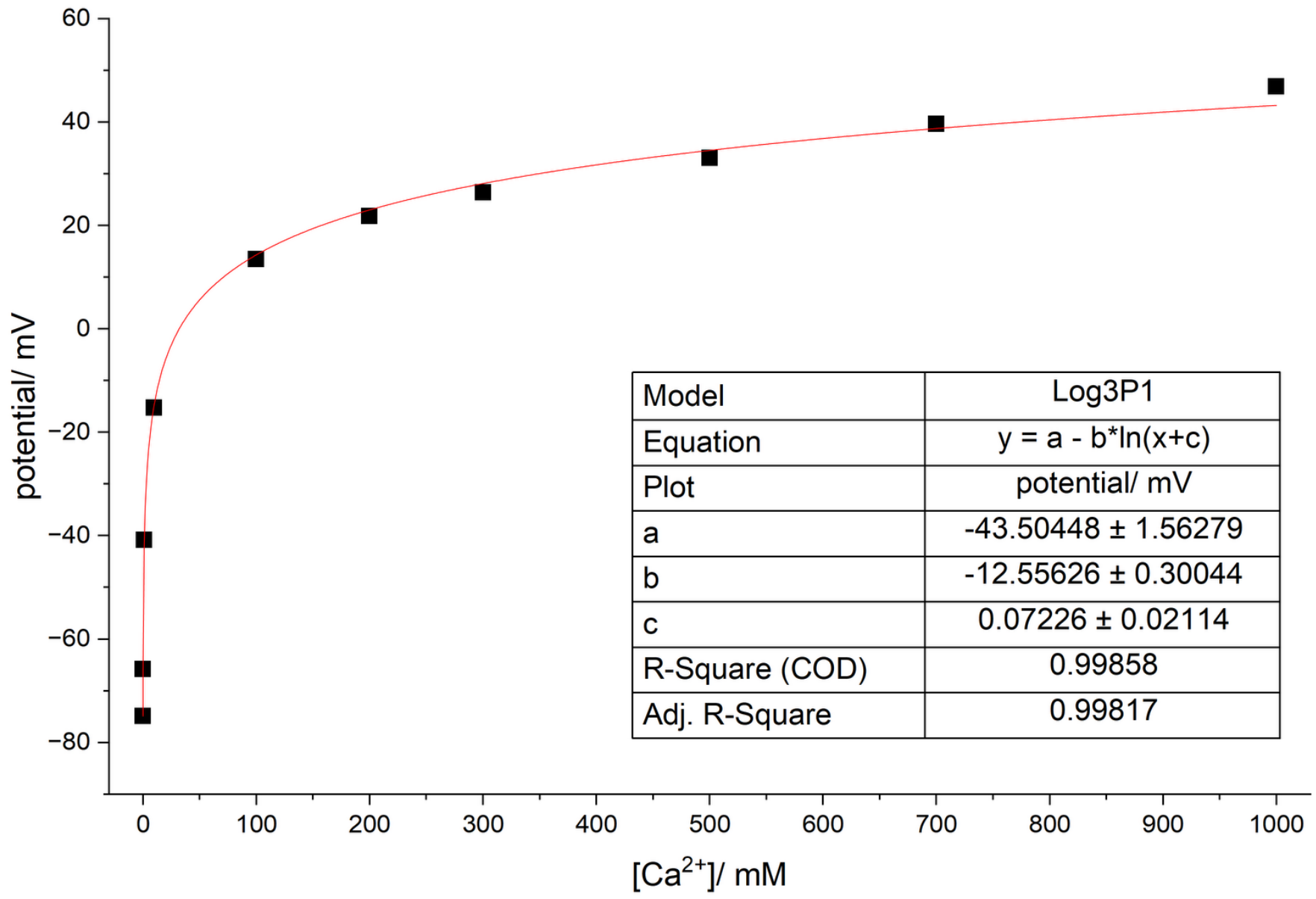

Figure S20: ISE calibration curve for Ca^{2+} standards.

Vesicle suspensions (1.5 mL, 1 mM lipid) were added to a 17 mm vial, and gently stirred. Transporter **2a** (1 mM or 0.5 mM) was added as a stock solution in DMSO at 60s, and voltage was monitored over time. At the end of the run (after 11 minutes), detergent (25 µL of Triton X-100 in 7:1 (v/v) H_2O-DMSO) is added to normalise the data. The data (potential, mV) was first converted to $[Ca^{2+}]$/ mM using equation S8. Subsequently, equation S9 was used to plot the data, where y_t is the calcium activity at time t, $y_0 = y_t$ before transporter addition, $y_{max} = y_t$ after lysis:

$$Calcium\ efflux = \frac{y_t - y_0}{y_{max} - y_0}$$

(Equation S9)

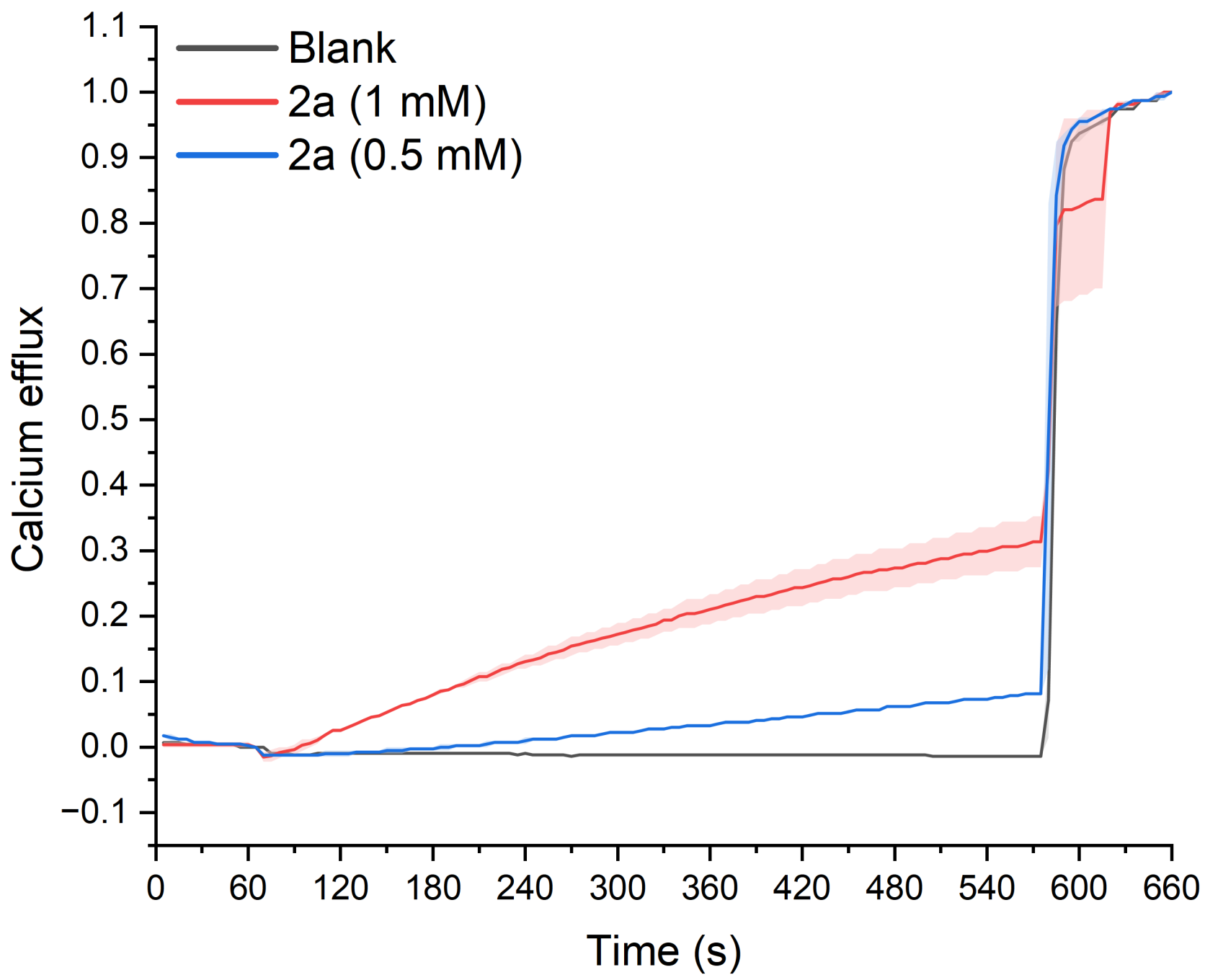

Figure S21: Measured Ca^{2+} efflux upon the addition of **2a** (1 mM or 0.5 mM) in DMSO in POPC LUVs (1 mM) containing 500 mM $CaCl_2$, 5 mM HEPES, pH 7.0, suspended in 500 mM NaCl, 5 mM HEPES, pH 7.0.

10. Single Liposome Calcium Transport Assay

The assay was performed following a modified version of a previously reported procedure.[3]

Vesicle preparation:

A thin lipid film was prepared by evaporating a chloroform solution of POPC (0.01 M) under reduced pressure on a rotavap to afford aliquots of 6 µmol. The films were further dried under high vacuum for 6 hours. The lipid films were hydrated with 500 µL of Fluo-8-containing buffer (10 µM Fluo-8, 10 µM EDTA, 20 mM HEPES, pH 7.0), sonicated for 30s, and vortexed for 1 hour. The resulting suspensions were subjected to 10 freeze-thaw cycles by alternating immersion in liquid nitrogen and a water bath (40 °C). The vesicles were then extruded 29 times through a polycarbonate membrane (pore size 200 nm). Extravesicular components were removed by size exclusion chromatography using an Izon qEV 70 nm column washed with buffer (20 mM HEPES, pH 7.0), yielding solution of vesicles with lipid concentrations of 2.5–4 mM. Lipid concentrations were determined by ^{1}H NMR spectroscopy.[4]

Microfluidic device preparation:

The microfluidic device design has been described previously,[5] and consists of a single channel (width = 100 µm, height = 25 µm, length = 1 cm). They were fabricated using standard soft-lithography techniques into polydimethylsiloxane (PDMS; Dow Corning) with SU-8 photoresist on silicon masters, as described previously.[6] The channels were oxygen plasma-bonded to glass coverslips (VWR, thickness = 1) to create sealed devices

Single-molecule confocal microscope:

The apparatus used for single molecule detection was similar to that previously described and shown in Figure S17.[7, 8] A Gaussian beam at 488 nm (LBX-405-100-CSB-OE, Oxxius) was directed through the back port of an inverted microscope (Nikon Eclipse TE2000-U), where it was reflected by a dichroic mirror (DI03-R405/488/561/635, Semrock) through an oil immersion objective (Nikon CFI Plan Apochromat VC 100x Oil, NA 1.4, W.D 0.13 mm) and focused 10 µm into the microfluidic device. Careful manipulation of the focal spot was used to find the position of vesicles flowing through the microfluidic device. Fluorescence was collected by the same objective and imaged onto a 50 µm pinhole (Thorlabs). The fluorescence was filtered by optical filters (long-pass: BLP01-488R-25 and band-pass: FF01-525/30-25, Semrock) before being focused onto an avalanche photodiode, APD (SPCM-14, Perkin Elmer, and Waltham, MA). Output from the APD was connected to a USB data acquisition card (USB-CTR04, Measurement Computing), which counted the signals and combined them into time-bins of 100 µs, the expected residence time of the vesicles in the confocal volume.

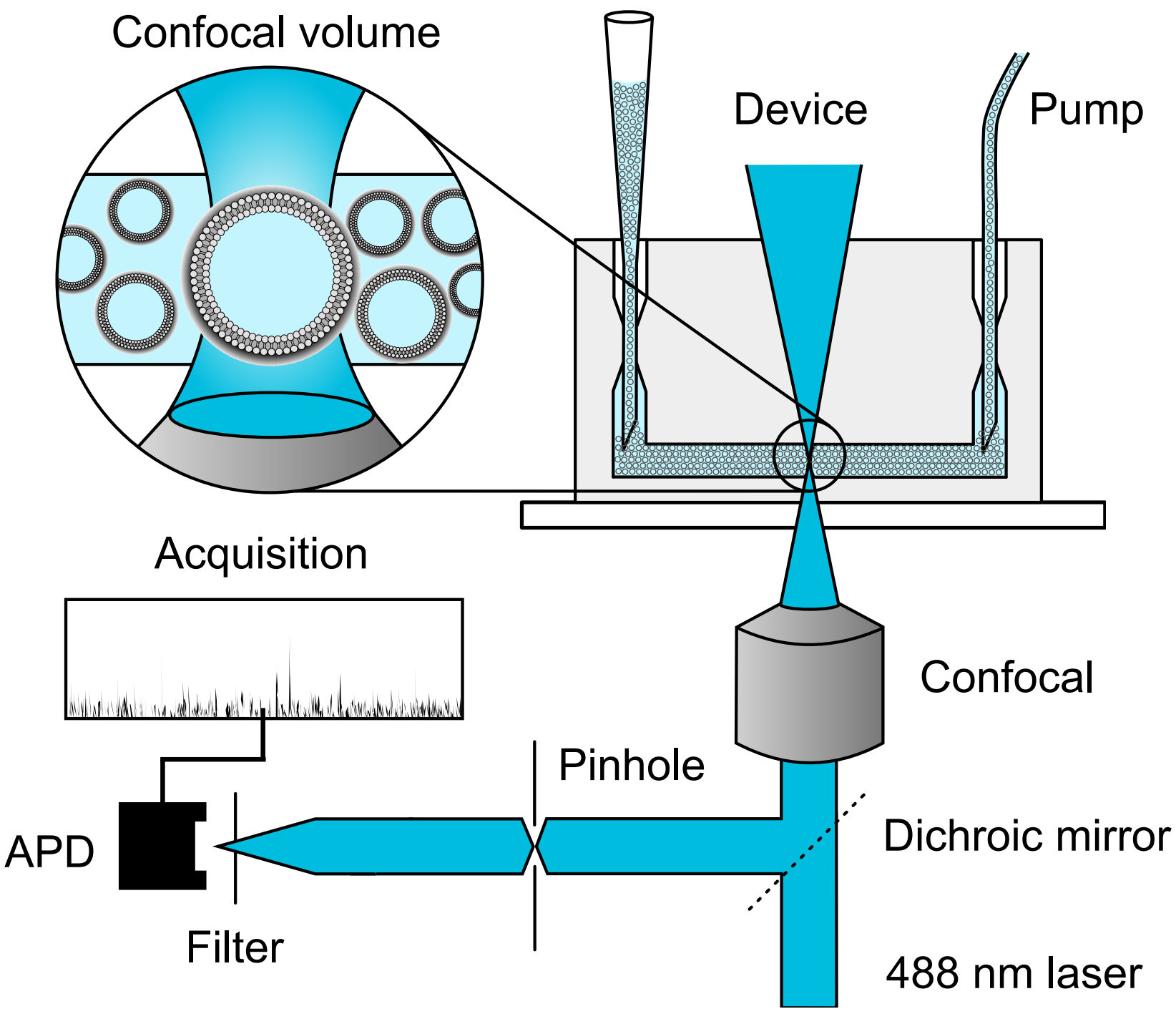

Figure S22. Schematic of the experimental setup, consisting of an inverted confocal fluorescence microscope interfaced with a flow cell. The microfluidic device was fabricated using standard soft lithographic methods.

<u>General procedure:</u>

Dye-filled vesicles were diluted with buffer (20 mM HEPES, pH 7.0) to a final lipid concentration of 0.005 mM. A 200 mM calcium chloride solution was prepared by dissolving $CaCl_2{\cdot}2H_2O$ in water. Stock solutions of permeabilising agents were diluted prior to analysis, and added to give final concentrations of 0.25–5 mol% relative to the lipid concentration. Ionomycin was diluted with buffer, while compound **2a** was diluted in DMSO.

For determination of I_{min} (the number of fluorescent events in a blank sample), 1 µL of $CaCl_2$ solution (200 mM) was added to 100 µL of dye-filled vesicles solution (0.005 mM lipid) and mixed gently. The sample was transferred in a gel-loading tip and mounted in a device. The microfluidic channel was initially washed with this solution at a flow rate of 1000 µL/hr for one minute, after which the flow rate was reduced to 96 µL/hr for one minute before measurements. Confocal measurements were acquired for 5 min at a sampling rate of 10,000 Hz using a 488 nm laser at 2.5 mW.

For determination of I_{max} (the number of fluorescent events in a fully permeabilised sample), 2 µL of ionomycin (2.5 µM) was added to 100 µL of dye-filled vesicles solution (0.005 mM lipid), followed by

1 µL of $CaCl_2$ solution (200 mM). The mixture was gently mixed and incubated at room temperature for 5 min before loading into the device and analysed under identical flow and acquisition conditions.

For determination of **2a** activity, 2 µL of **2a** (12.5–0.625 µM) was added to 100 µL of dye-filled vesicles solution (0.005 mM), followed by 1 µL of $CaCl_2$ solution (200 mM). After gentle mixing and incubation for 5 min at room temperature, samples were analysed using the same flow and confocal measurement conditions described above.

To minimise variation between vesicle batches, event counts were normalised using the I_{min} and I_{max} measurements. I values were obtained by analysing raw confocal data files in which individual photon bins were assessed using a predefined intensity threshold (typically 60 photons/s). I corresponds to the number of bins exceeding this threshold during the 5 min acquisition period. The I_{min} value corresponds to the number of events in a blank sample containing Ca^{2+} (2 mM) and dye-loaded vesicles (0.005 mM, lipid concentration). The I_{max} value indicates the number of events corresponding to fully sensitised dye signal, which is determined by the Ca^{2+} permeabilisation with ionomycin (1 mol% to lipid). The number of events from the analysed sample (I_{sample}), I_{min}, and I_{max} produced normalised value of activity (I_{rel}) with equation:

$$I_{rel} = \frac{I_{sample} - I_{min}}{I_{max} - I_{min}}$$

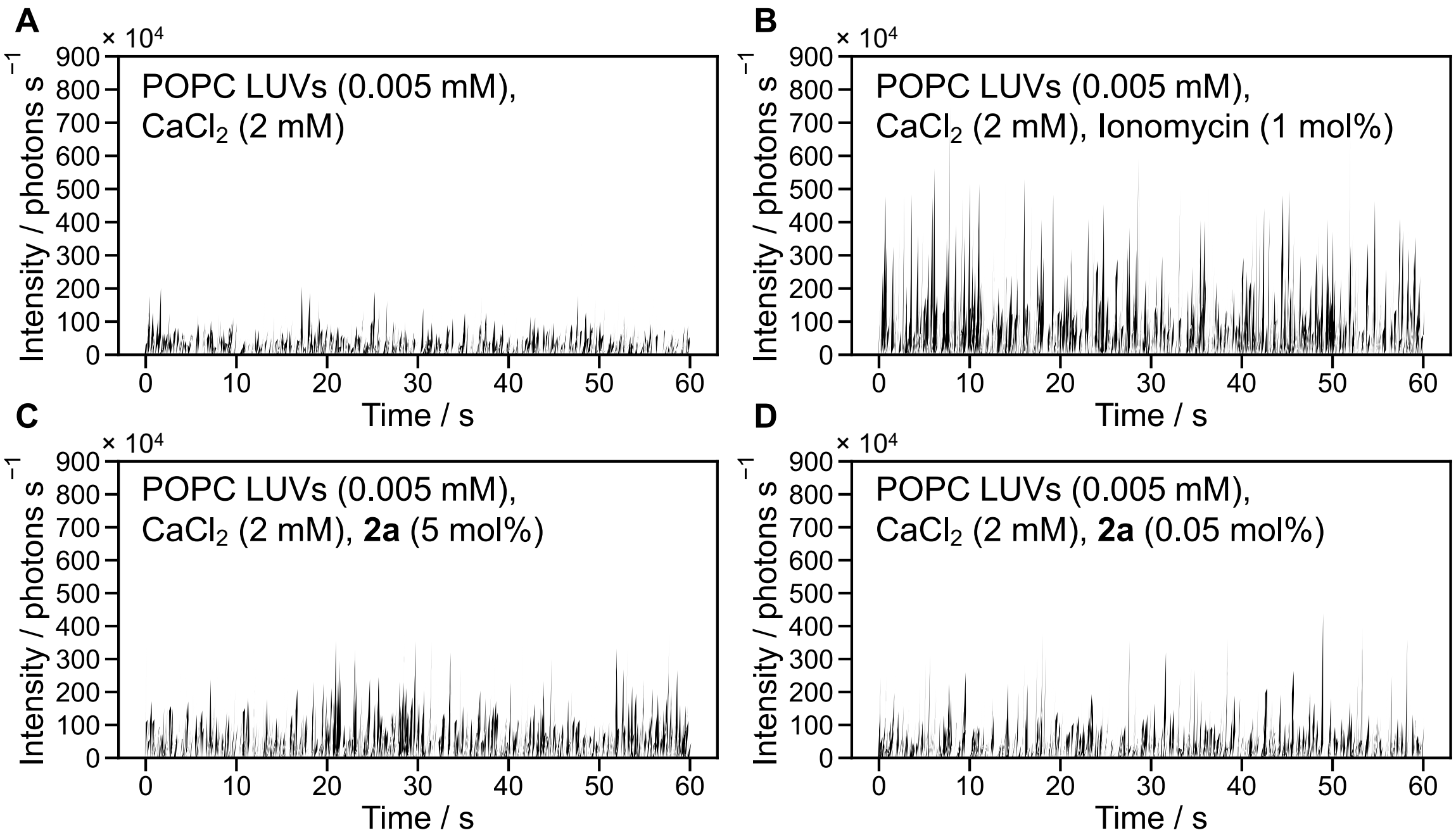

Figure S23. Representative calcium transport studies with ionomycin and **2a**. **A**. Example of data collected over one minute for Fluo-8 dye-filled LUVs in the presence of $CaCl_2$ (to calculate I_{min}). **B**. Example of data collected over one minute for Fluo-8 dye-filled LUVs in the presence of $CaCl_2$ and ionomycin (to calculate I_{max}). **C-D**. Example data collected over one minute for Fluo-8 dye-filled LUVs in the presence of $CaCl_2$ and varying concentrations of **2a**.

11. References

1 I. V. Tetko, J. Gasteiger, R. Todeschini, A. Mauri, D. Livingstone, P. Ertl, V. A. Palyulin, E. V. Radchenko, N. S. Zefirov, A. S. Makarenko, V. Y. Tanchuk and V. V. Prokopenko, *J. Comput. Aided Mol. Des.*, 2005, **19**, 453–463.

2 K. K. Behara, Y. Rajesh, Y. Venkatesh, B. R. Pinninti, M. Mandal and N. D. P. Singh, *Chem. Commun.*, 2017, **53**, 9470–9473.

3 K. M. Bąk, D. C. Edwards, D. George, B. Singh, R. Ferguson, T. Zhao, K. Piché, A. Louwrier, S. L. Cockroft and M. H. Horrocks, *Angew. Chem. Int. Ed.*, 2025, **e202503678**.

4 R. Hein, C. B. Uzundal and A. Hennig, *Org. Biomol. Chem.*, 2016, **14**, 2182–2185.

5 M. H. Horrocks, L. Tosatto, A. J. Dear, G. A. Garcia, M. Iljina, N. Cremades, M. Dalla Serra, T. P. J. Knowles, C. M. Dobson and D. Klenerman, *Anal. Chem.*, 2015, **87**, 8818–8826.

6 M. H. Horrocks, H. Li, J.-u. Shim, R. T. Ranasinghe, R. W. Clarke, W. T. S. Huck, C. Abell and D. Klenerman, *Anal. Chem.*, 2012, **84**, 179–185.

7 A. Orte, R. Clarke, S. Balasubramanian and D. Klenerman, *Anal. Chem.*, 2006, **78**, 7707–7715.

8 A. Chappard, C. Leighton, R. S. Saleeb, K. Jeacock, S. R. Ball, K. Morris, O. Kantelberg, J. E. Lee, E. Zacco, A. Pastore, M. Sunde, D. J. Clarke, P. Downey, T. Kunath and M. H. Horrocks, *Angew. Chem. Int. Ed.*, 2023, **e202216771**.
